# In Vitro Cytotoxicity, Colonisation by Fibroblasts and Antimicrobial Properties of Surgical Meshes Coated with Bacterial Cellulose

**DOI:** 10.3390/ijms23094835

**Published:** 2022-04-27

**Authors:** Karolina Dydak, Adam Junka, Grzegorz Nowacki, Justyna Paleczny, Patrycja Szymczyk-Ziółkowska, Aleksandra Górzyńska, Olga Aniołek, Marzenna Bartoszewicz

**Affiliations:** 1 Department of Pharmaceutical Microbiology and Parasitology, Wroclaw Medical University, Borowska 211a, 50-556 Wroclaw, Poland; justyna.paleczny@student.umw.edu.pl (J.P.); marzenna.bartoszewicz@umw.edu.pl (M.B.); 2 Department of Laboratory Diagnostics Health Care Complex in Nysa, Bohaterów Warszawy 23, 48-300 Nysa, Poland; grzesiek.nowacki95@gmail.com; 3 Centre for Advanced Manufacturing Technologies (CAMT/FPC), Faculty of Mechanical Engineering, Wroclaw University of Science and Technology, Łukasiewicza 5, 50-371 Wroclaw, Poland; patrycja.e.szymczyk@pwr.edu.pl; 4 Student Research Circle, Department of Pharmaceutical Microbiology and Parasitology, Wroclaw Medical University, Borowska 211a, 50-556 Wroclaw, Poland; ola.gorzynska@wp.pl; 5 Faculty of Medicine, Lazarski University, 02-662 Warsaw, Poland; olga.aniolek@lazarski.pl

**Keywords:** hernia mesh, bacterial cellulose, gentamicin, biocompatibility

## Abstract

Hernia repairs are the most common abdominal wall elective procedures performed by general surgeons. Hernia-related postoperative infective complications occur with 10% frequency. To counteract the risk of infection emergence, the development of effective, biocompatible and antimicrobial mesh adjuvants is required. Therefore, the aim of our in vitro investigation was to evaluate the suitability of bacterial cellulose (BC) polymer coupled with gentamicin (GM) antibiotic as an absorbent layer of surgical mesh. Our research included the assessment of GM-BC-modified meshes’ cytotoxicity against fibroblasts ATCC CCL-1 and a 60-day duration cell colonisation measurement. The obtained results showed no cytotoxic effect of modified meshes. The quantified fibroblast cells levels resembled a bimodal distribution depending on the time of culturing and the type of mesh applied. The measured GM minimal inhibitory concentration was 0.47 µg/mL. Results obtained in the modified disc-diffusion method showed that GM-BC-modified meshes inhibited bacterial growth more effectively than non-coated meshes. The results of our study indicate that BC-modified hernia meshes, fortified with appropriate antimicrobial, may be applied as effective implants in hernia surgery, preventing risk of infection occurrence and providing a high level of biocompatibility with regard to fibroblast cells.

## 1. Introduction

Surgical meshes are implants ensuring the proper structure of organs and tissues within a patient’s body. The main indications for the use of surgical mesh are mainly cases of abdominal hernia or reconstruction of the oesophagus [1,2]. The use of surgical mesh seems to be indispensable in these types of procedures, but their proper management is still the subject of ongoing debates [3]. The application of surgical mesh seems to be effective even in complicated cases referred to incarcerated or strangulated hernias [4].

The use of surgical meshes to reinforce anatomical structures started already in the 1950s. The first products of this kind were stable constructions made of polypropylene, but their application was frequently associated with scarring and irritation [5]. Presently, the materials (of synthetic or animal origin) of which hernia meshes consist must display high biocompatibility and durability. The meshes can be generally divided into non-absorbable (permanent) and those that are gradually resorbed after implantation. The meshes of the latter are not intended to provide permanent reinforcement but to allow the tissue to grow over and to recreate their own structure. Such absorbable meshes are commonly obtained from animal-derived material (such as pig or cattle skin and intestines), which requires specific processing and sterilization before implantation [6]. There is also another type of mesh in which dissolvable and permanent materials are applied together. Such an approach combines the desired material properties of synthetic polymers together with the low (to none) level of cytotoxicity displayed by specific natural polymers. Appropriate examples of such a biocompatible coating are carboxycellulose gel and fibroblast cell layers. To decrease the risk of surgical site infection (SSI), such natural coatings are often chemisorbed or saturated with various antimicrobial agents [7,8,9]. The main problem with the use of surgical mesh is postoperative infective complications, which may lead to general infection (sepsis). According to the review data, the risk of mesh-related infection in hernia operations ranges from several to even 10% [10,11], which corresponds to at least several tens of thousands of cases annually requiring complex, expensive treatment and often to the necessity of hernia removal [10,12]. The microorganisms contaminating the implanted surgical mesh are mostly of endogenic origin and they transfer to the mesh from the patient’s oral cavity, skin or gut [13]. The main factors of infection are of mainly bacterial origin (with such microorganisms involved as *Staphylococcus aureus*, *Streptococcus* spp., *Enterobacteriaceae*, *Peptostreptococcus anaerobes*); fungus-related infections are of less frequent character [14,15]. The adhesion of microorganisms to the hernia surface may lead to biofilm development and to further, distinct complications (with regard to the time of manifestation). To counteract the risk of infection emergence, the development of new, effective antimicrobial mesh adjuvants is required. In many cases, surgeons face a serious problem of mesh presence-related infections. Maintaining appropriate microbiological antisepsis of the surgical site and reduction of surgery time are factors decreasing the infection risk [16]. There are also reports indicating that the use of laparoscopic methods can reduce the complications related to infections, but only to a level of about 0.7–2% [15]. Nevertheless, infection-related complications may require the reoperation of the patient, including removal of the mesh [17,18,19]. Therefore, the use of appropriate prophylaxis seems indispensable to minimize the costs of treatment as well as the unnecessary difficulties to which the patient is exposed. In hernia surgery, saturation of the mesh with antibiotics before implantation is becoming more and more common in order to minimize the risk of infection [20].

Bacterial cellulose (BC, bionanocellulose) is a polymer produced by numerous genres of bacteria, including *Komagataeibacter*, *Aerobacter*, *Azotobacter*, *Alcaligenes*, *Achromobacter*, *Pseudomonas*, *Agrobacterium*, *Burkholderia*, *Dickeya*, *Rhizobium*, *Sarcina*, *Enterobacter*, *Salmonella* and *Escherichia* [21]. The Gram-negative *Komagataeibacter xylinus* is considered an example of a microorganism best suited for bacterial cellulose production by fermentation [22]. It produces a hydrated, flexible membrane. Chemically, BC is a polymer having the structure of linear beta-1,4-glucan chains. It consists of randomly connected ribbon-shaped fibres < 100 nm wide, which in turn consist of smaller nanofibers 7–8 nm wide aggregated into bundles. Its significant advantage is that it does not require complex purification procedures in the industrial process. In contrast to plant cellulose, bacterial cellulose is also free of impurities such as hemicellulose, waxes, pectin or lignin. Additionally, it has a higher level of crystallinity and polymerization [23,24]. The molecules forming BC form a structural, spatial, three-dimensional network, which determines the durability and flexibility of this material. The advantages of bacterial cellulose also include the fact that it does not cause toxic effects on cells and tissues of the human body [25,26,27,28]. It has a high water absorption capacity and is completely biodegradable and safe for the environment [29]. The properties of the produced BC depend on various culture conditions (including duration of culture, medium composition and BC purification methods) [21,22].

Currently, BC is used in many industrial fields, such as cosmetics [30,31], papermaking and preserving [32,33], the textile industry [34,35], environmental protection [36,37], the electrotechnical industry [38,39], OLED diodes production [40,41], the food industry [42,43], scientific research [44,45,46,47] and medicine. Medical applications of BC include, for example, dressings, artificial muscles, skin and blood vessels, cartilage and dental implants [48,49,50,51].

In order to improve the properties of BC, i.e., increase its strength and elasticity, it is saturated with various substances, e.g., alginate, collagen, chitosan or polyphosphates. The properties of BC also make it suitable for use as a drug carrier. This makes BC an ideal material for use as an effective dressing against bacteria (also in the form of a biofilm). At the same time, it is suitable for implementation inside the patient’s body and tissues thanks to its negligible toxicity and high biocompatibility [52].

To date, a number of trials have been conducted to evaluate the effectiveness of surgical mesh made of bacterial cellulose. There are animal studies indicating these meshes’ high biocompatibility and lack of sensitization or inflammatory reaction [53]. There are also studies concerning the effective coating of surgical mesh with bacterial cellulose. Ludwicka et al. focused on methods of coating the meshes with BC and on cell cytotoxicity and degranulation assays in short duration experiments (24 h). The authors did not assume in their studies an evaluation of the BC fortified with antimicrobials [54], while such a solution ensures the combination of durability of propylene material with good parameters of cellulose biocompatibility together with the infection risk-minimizing activity of antimicrobials.

Therefore, the aim of our investigation was to evaluate the suitability of bacterial cellulose coupled with antibiotics as an absorbent layer of surgical mesh in the context of antimicrobial prophylaxis. We also carried out a long-term cell colonisation measurement assay (60 days) to investigate if the high level of BC-modified mesh colonisation by fibroblasts, reported in other research, are maintained during such a long period of time.

## 2. Results

### 2.1. Sample Preparation

**Figure 1:**
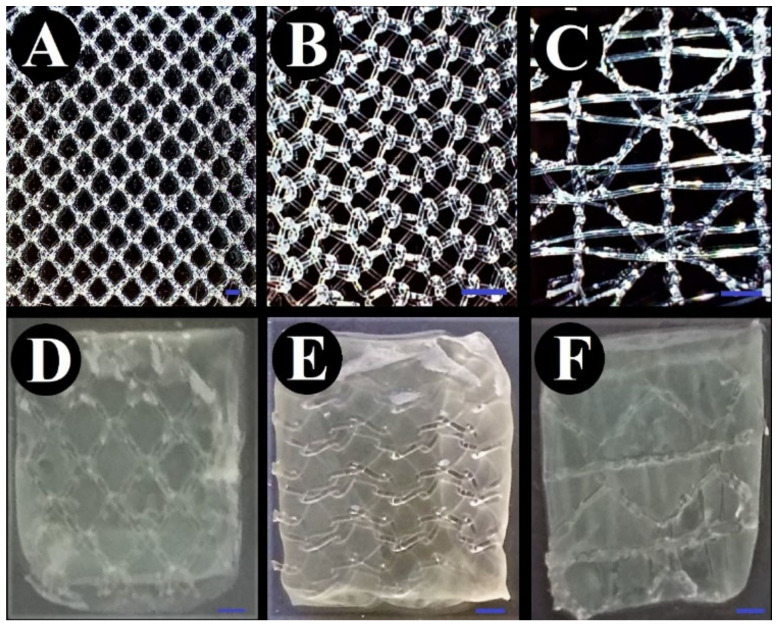
Bacterial cellulose-coated and uncoated surgical meshes. (**A**–**C**) uncoated meshes, (**D**–**F**) bacterial cellulose-coated meshes; (**A**,**D**)—M1; (**B**,**E**)—M2; (**C**,**F**)—M3, the blue bar represents the length of 2 mm.

The applied meshes displayed statistically different sizes of pores (*p* < 0.0002) as presented in Table S2 in Supplementary Materials. The coating meshes with BC lasted for 6 days. The effect of this process is shown in Figure 1.

### 2.2. Cytotoxicity ASSAY

**Figure 2:**
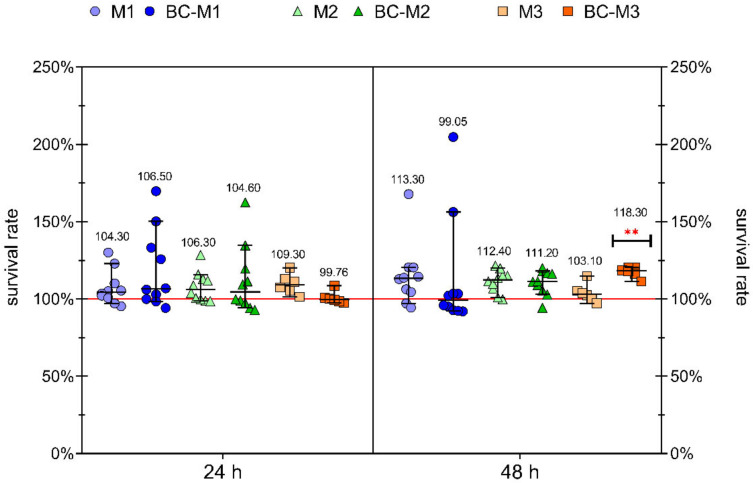
Survival rate (%) of fibroblast cell line ATCC CCL-1 with 24 h and 48 h extracts from bacterial cellulose-coated and uncoated surgical meshes. M1, M2, M3—uncoated meshes; BC-M1/M2/M3—meshes coated with bacterial cellulose; whiskers show median with 95% confidence interval; median values (%) are shown over every scatter dot plot; ** moderate statistically significance (*p* = 0.0059); red line is control sample = 100% of survivability.

To evaluate if bacterial cellulose does not have toxic effects on fibroblast cells, a normative cytotoxicity assay was performed. Two types of extracts (obtained after immersion of samples for 24 h and 48 h contact time) were tested. Results are shown in Figure 2. No significant differences between M1, M2 and M3 meshes (regardless if they were BC-coated or not) and native (control, uncoated) meshes were observed (K–W test, α = 0.05), with the single exception observed for fibroblasts treated with 48 h extract from BC-M3 mesh compared to control samples (fibroblasts ATCC CCL-1 incubated with fresh culture medium DMEM; *p* = 0.0059). For extracts obtained from 10/12 of the treated samples, higher survival rates than in control sample were observed. The other two out of twelve demonstrated a very slight decrease of this parameter (median of survival rate = 99.76% and 99.05% for 24 h extract from BC-M3 and 48 h extract from BC-M1, respectively). The highest survivability showed fibroblasts treated with 48 h extract from BC-M3 (median of survival rate = 118.30%). The higher average survival rate was observed for fibroblasts treated with 48 h extracts than 24 h (109.56% and 105.13%, respectively). The detailed statistical data (Table S3) and graphical demonstration of results (Figure S1) are shown in the Supplementary Materials.

### 2.3. Cell Colonisation Measurement

The measurement of mesh colonisation by cells was carried out for 60 days. Three types of surgical meshes with and without BC were tested. A total of 15 series of measurements was taken on the 4th, 8th, 12th, 16th, 20th, 24th, 28th, 32nd, 36th, 40th, 44th, 48th, 52nd, 56th and 60th day of culture. For every sample, the significant increase of cell quantity between the first and the last measurement was observed, with the exception of the BC-M1 sample (Table S4 in Supplementary Materials).

In all samples, an increase of fibroblast quantity to the 28th or 32nd day of culture was observed, and then a decrease was noticed. The results obtained for samples M1 and BC-M1 were differentiated from the remaining results. Fibroblast growth on the M1 was very poor during the entire duration of culturing. The absorbance value between the 4th and 60th day of culture increased less than 6 times (about 15 and 20 times for M2 and M3, respectively). The first growth peak was observed on the 32nd day of culture, and then it decreased, and there was another increase on the 44th day of culture. Between the 44th and 56th day of culture, the fibroblasts growth was on a constant level, and on the 60th day of culture a moderate increase was observed. In the last day of culture, the highest quantities of fibroblasts on the M1 surface were observed. Sample BC-M1 demonstrated a different pattern of fibroblasts growth. The first growth peak was observed on the 12th day of culture; then there was a slight decrease and another peak on the 20th day of culture. Then, two periods, namely a decrease and increase of fibroblast quantity was noticed. The highest quantity of fibroblasts was measured on the 20th day of culture. The high decrease of fibroblasts quantity was observed on the 40th day of culture. Comparing fibroblast growth on M1 and BC-M1 samples, there were very noticeable differences in favour of the BC-M1 sample. On each measurement day between the 4th and 32nd, the differences between M1 and BC-M1 samples were significant (*p* < 0.0001), and later the differences decreased (no significant difference on days 40th, 52nd and 60th; *p* = 0.0130 on 48th day, and *p* = 0.0095 and *p* = 0.0091 on the 48th and 56th days, respectively). More statistical details and graphical demonstrations of results are shown in Figures S2 and S3 and in Table S5 in Supplementary Materials.

Fibroblast quantity on the M2 sample increased until the 32nd day of culture and then decreased, and another increase was observed on the 48th and 60th days. The highest fibroblast quantity on the M2 sample was noticed on the 32nd day of culture. The BC-M2 sample showed an increase of fibroblasts quantity to the 28th day with an additional peak on the 12th day of culture. Then a decrease was noticed to the 44th day and a second peak on the 48th day. The highest fibroblast quantity was observed on the 48th day. In most measurement points, the fibroblasts quantity on the BC-M1 sample was higher than on the M1 sample, except on days 32, 40, 44 and 60. The differences between coated and uncoated surgical meshes were lower than for samples M1 and BC-M1. Only two measurement points showed statistical significance—these were day 12 and 16 (*p* < 0.0001 and *p* = 0.0002, respectively). Statistical data and graphical demonstration of results for samples M2 and BC-M2 are shown in Figures S4 and S5 and in Table S6 in Supplementary Materials.

The M3 sample represented a very similar pattern of fibroblasts growth to the pattern observed when M2 mesh was applied as the culture surface. There were also three peaks of growth on the 32nd, 48th and 60th days of culture, and the highest fibroblast quantity was noticed on the 32nd day. Growth of fibroblasts on the BC-M3 sample resembled also the growth pattern observed when BC-M2 mesh was applied, i.e., the increase of fibroblast growth to the 28th day with an additional peak on the 12th day of culture. Then a decrease was noticed on the 44th day and a second peak on the 48th day. The highest quantity of fibroblasts was observed on the 32nd day of culture. In contrast to M2 and BC-M2 samples, there were higher fibroblast quantities on sample BC-M3 than on sample M3, except on the 60th day of culture. Similarly to M2 and BC-M2 samples, significant differences were observed on the 12th and 16th day (*p* < 0.0001 and *p* = 0.0108, respectively). More statistical details and graphical demonstrations of results for samples M3 and BC-M3 are shown in Figures S6 and S7 and in Table S7 in Supplementary Materials.

**Figure 3:**
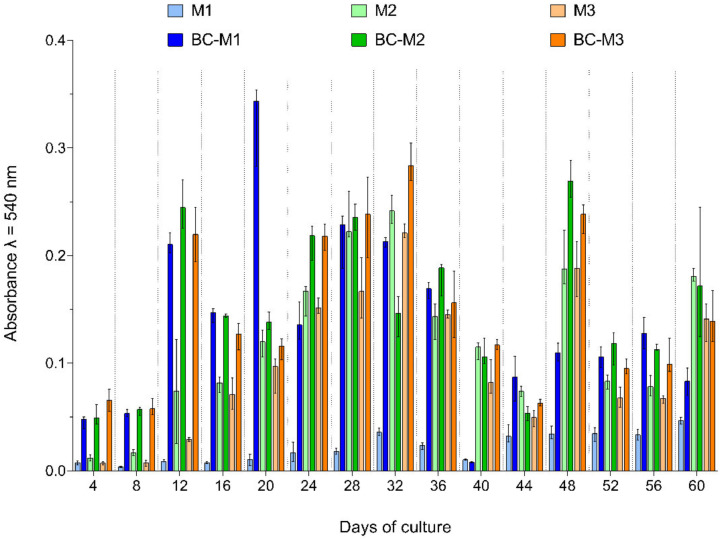
Graphical demonstration of colonisation of bacterial cellulose-coated and uncoated surgical meshes by fibroblast cell line ATCC CCL-1 during 60-day culture. M1, M2, M3—uncoated meshes; BC-M1/M2/M3—meshes coated with bacterial cellulose; whiskers show median with 95% confidence interval.

Graphical demonstration of summary results for all samples are shown in Figure 3.

Comparing uncoated samples M1, M2 and M3, the difference in fibroblast quantities between M1 and the rest of samples was noticeable. The average fibroblast quantity between M2 and M1 differed by 5.93 times (SD = 3.14, ME = 5.36, range 1.68–10.61), and between M3 and M1 by 4.65 times (SD = 2.68, ME = 4.39, range 0.92–9.04). The average difference between M2 and M3 samples was low in comparison to the data shown above, c.a. 1.38 times (SD = 0.49, ME = 1.22, range 0.97–2.89). The differences in fibroblast quantity between M1 and the rest of samples were significant (*p* < 0.0001 and on the 60th day between M1 and M3, *p* = 0.0006) in almost all measurement points, except the 4th, 8th, 12th, 44th, 52nd and 56th day. On the 12th day of culture, only differences between M2 and M1 samples were significant (*p* < 0.0001). Between M2 and M3 samples there were no significant differences. More statistical details and graphical demonstrations of results for uncoated meshes are shown in Figure S8 and in Table S8 in Supplementary Materials.

The changes in fibroblast quantities observed in bacterial cellulose-coated meshes displayed the more similar trend between themselves than was observed when uncoated meshes were applied as growth surfaces. The average fibroblast quantity was 2.085 times higher (SD = 3.57, ME = 1.07, range 0.4–14.85) on BC-M2 and 2.091 times higher (SD = 3.63, ME = 1.07, range 0.36–15.11) on BC-M3 than on BC-M1. The average difference between BC-M2 and BC-M3 samples was almost negligible and was about 1.01 times (SD = 0.20, ME = 1.04, range 0.55–1.34). Differences between fibroblast quantities between BC-M1 and the rest of samples were (*p* < 0.0001) significant only on three measurement points—on the 20th, 40th and 48th days of culture. On the 24th and 60th days there were significant differences only between BC-M1 and BC-M3 samples (*p* = 0.0382 and *p* < 0.0001, respectively). Significant differences between BC-M2 and BC-M3 samples were observed only on the 32nd day of culture (*p* = 0.0016). More statistical details and graphical demonstrations of results for uncoated meshes are shown in Figure S9 and in Table S9 in Supplementary Materials.

Considering fibroblast quantity during the whole culture period, the distribution of cells in measurement days resembles a bimodal distribution with additional peaks. For all samples, the first peak of growth was on the 28th or 32nd day and the second one on the 56th or 60th day. The additional growth peaks for uncoated samples were on the 48th day of culture, except the M1 sample, which showed no additional peak. For all BC-coated samples, the first additional growth peak was on the 12th day of culture, and the second for the BC-M1 sample was on 20th day, and for BC-M2 and BC-M3 samples, they were on the 48th day of culture. 

**Figure 4:**
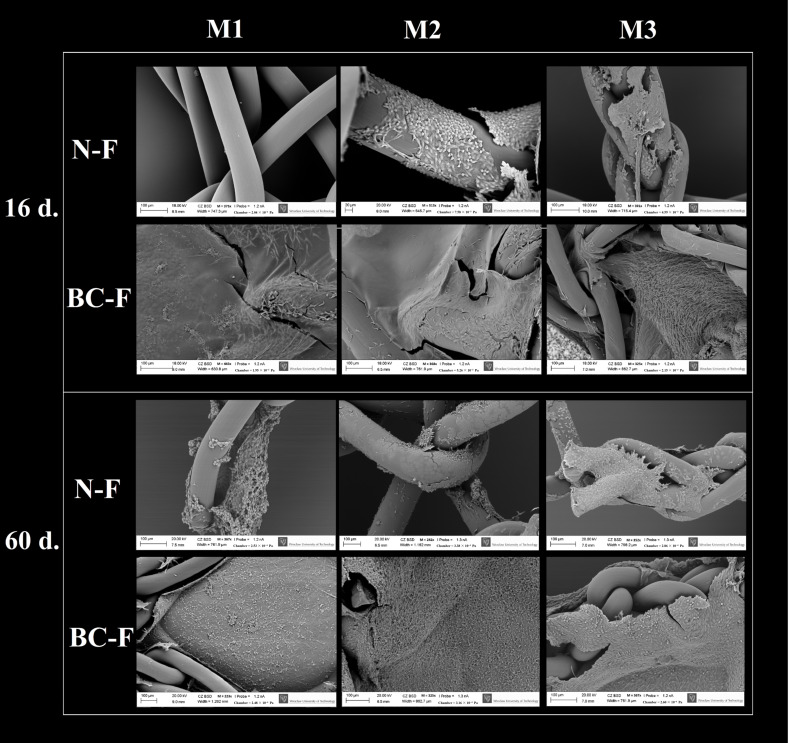
SEM images showing changes in cell quantity between the 16th and 60th day of culture. M1, M2, M3—tested meshes; N-F—native meshes with fibroblasts; BC-F—bacterial cellulose-coated meshes with fibroblasts; 16 d.—16th day of culture; 60 d.—60th day of culture scanning electron microscope (SEM, Zeiss EVO MA25, Oberkochen, Germany); M—magnification (223×–513×, below every picture).

SEM images showing changes in cell quantity between the 16th and 60th day of culture on BC-coated and uncoated meshes are shown in Figure 4.

### 2.4. Determination of Minimal Inhibitory Concentration (MIC) and Minimal Biofilm Eradication Concentration (MBEC) of Gentamicin

**Figure 5:**
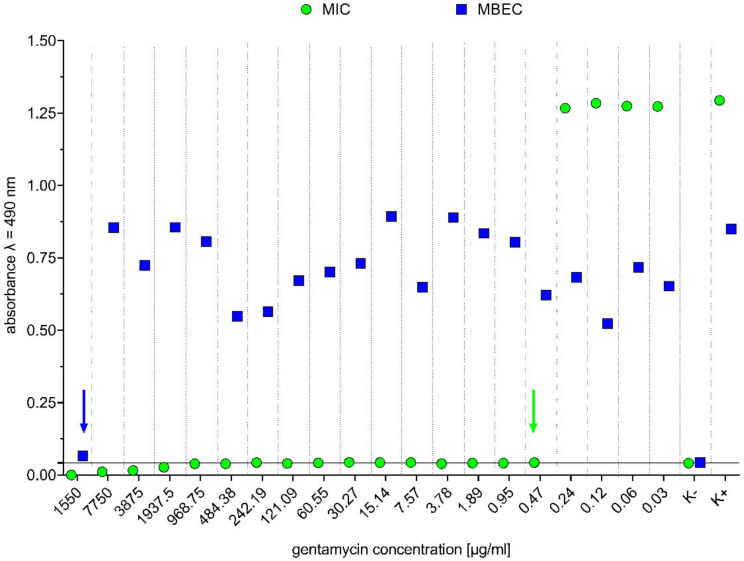
Minimal inhibitory concentration (MIC) and minimal biofilm eradication concentration (MBEC) of gentamicin for *Staphylococcus aureus* ATCC 33591. Green arrow indicates MIC value (0.47 µg/mL), and blue arrow indicates MBEC value (1.55 mg/mL). K– is a negative control, and K+ is positive.

In MIC and MBEC tests, a wide range of gentamicin concentrations was examined (from 1.55 mg/mL to 0.03 µg/mL). For *Staphylococcus aureus* ATCC 33591, the MIC of gentamycin was 0.47 µg/mL and that of MBEC was 1.55 mg/mL. Results are shown in Figure 5.

### 2.5. Bacterial Cellulose Water Content Determination

**Figure 6:**
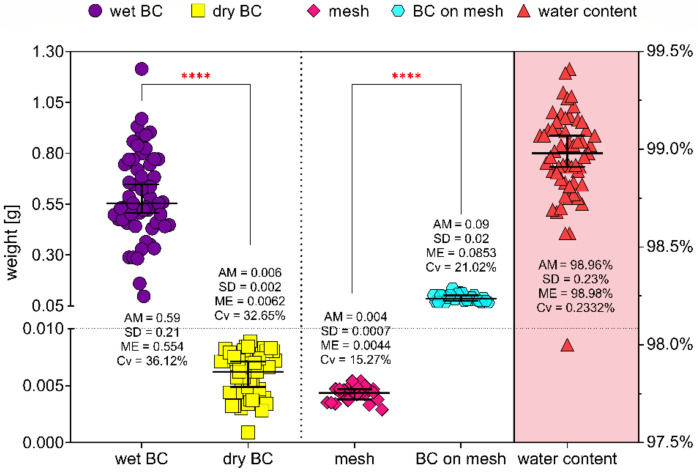
Graphical demonstration of meshes and bacterial cellulose weight and water capacity of bacterial cellulose. AM—arithmetic mean; SD—standard deviation; ME—median; Cv—coefficient of variation; ****—very high statistically significance (*p* < 0.0001); whiskers show median with 95% of confidence interval.

The total number of 24 BC-coated meshes, 24 uncoated meshes and 57 BC discs were weighed. The water capacity of bacterial cellulose was calculated at about 99%. Between wet and dry BC discs and BC-coated and uncoated meshes, statistically significant differences were observed (*p* < 0.0001). Results are shown in Figure 6, and additional statistical data are in Table S10 in Supplementary Materials.

### 2.6. Modified Disc Diffusion Method

**Figure 7:**
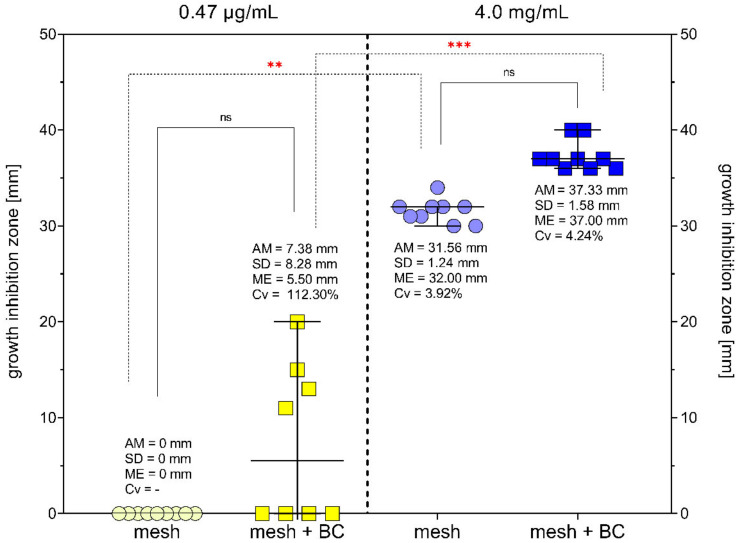
*Staphylococcus aureus* ATCC 33591 growth inhibition zones (mm^2^) caused by bacterial cellulose-coated and uncoated meshes, saturated with gentamicin in two concentrations: 0.47 µg/mL and 4.0 mg/mL. AM—arithmetic mean; SD—standard deviation; ME—median; Cv—coefficient of variation; ***—high statistically significance (*p* = 0.0003); **—moderate statistically significance (*p* = 0.0084); ns—no significant differences; whiskers show median with 95% of confidence interval.

To evaluate antimicrobial activity of BC-coated and uncoated meshes, the growth inhibition zones were measured. Results are shown in Figure 7.

Two concentrations of gentamicin were tested—0.47 µg/mL, which was determined as minimal inhibitory concentration against *S. aureus*, and 4.0 mg/mL, which is the concentration applied in the commercially available product, referred to as the gentamycin sponge. The profiles of gentamicin released from the coated and uncoated meshes are presented in Figures S10 and S11 in the Supplementary Materials. The application of meshes coated with BC and saturated with 0.47 µg/mL of gentamicin was translated into the bacterial growth inhibition zone in the applied experimental model (meshes without BC did not inhibit bacterial growth). The growth inhibition zones were of higher size when the higher (4.0 mg/mL) concentration of gentamycin was applied. More statistical data are shown in Table S11 in Supplementary Materials.

## 3. Discussion

The first surgical meshes were used already in the 1950s. According to the Global Alliance for Infections in Surgery, hernia repairs are the most common elective abdominal wall procedures performed by general surgeons. The infections related with mesh presence occur with 10% frequency, and they are considered the most deleterious complication of the operation. They require prolonged hospitalizations and often mesh removal [11,55]. To improve standards of treatment, new concepts related with modification of mesh shapes, structures and composition are currently developed. The research focuses on enhancement of meshes’ mechanical strength, biocompatibility and provision of antimicrobial agents in order to accelerate the healing, increases in cell colonization and reduction of the infection risk occurrence. The application of bacterial cellulose in the character of self-reliant hernia mesh or mesh coating fits, to the greatest extent, in these trends. 

Therefore, the aim of our research was to answer two questions—firstly, does the addition of BC have a positive effect on the mesh colonization by fibroblasts, and secondly, is the addition of an antimicrobial substance to the BC-coated mesh a sufficient measure to inhibit the growth of *S. aureus*, one of the most notorious opportunistic pathogens. Three different surgical meshes were applied for these purposes. In all three of them, the polypropylene was a basal material, but they differed with regard to the pore size and distribution. The survival rate of fibroblasts was over 99% for all tested meshes (native and BC-coated ones), proving lack of their in vitro cytotoxicity (Figure 2). These observations are consistent with the results from our previous research in which we showed that BC alone or applied as the coating of the prototypic orthopaedic implant does not induce any cytotoxic effect towards osteoblast and fibroblast cell lines [56,57]. In our previous work we also showed that fibroblasts, introduced to the BC surface in inoculum of 10^5^ cells/mL, multiplied in the undisturbed manner till they reached confluence in the 7th day of the experiment [58]. The fact of lack of BC cytotoxicity is a generally recognized phenomenon and was already confirmed by the number of other research teams. As an example, Jeong et al. evaluated the toxicity of BC in vitro using human umbilical vein endothelial cells and in an in vivo mouse model [28]. Research has shown that BC presence did not lead to the alterations of cell morphology, apoptosis or necrosis of scrutinized cells. The outcomes of the in vivo analysis revealed also no adverse effects with regard to body and organ weight of tested animals [28]. Kim et al. conducted similar in vitro and in vivo research on the same cell type. Their results also proved the lack of apoptosis or necrosis caused by BC. Moreover, this polymer did not affect T-cell differentiation and production of inflammatory mediators (IL-4, IFN-γ and COX-2) in both in vitro and in vivo models [59]. Volova et al. showed that BC did not cause cytotoxicity upon direct contact with fibroblasts. Contrary, it enabled high survival of the cells [60]. Goldschmidt et al. evaluated the influence of BC on dural fibroblasts in an in vitro study. The results of a 4 week-long experiment showed that fibroblasts penetrated the BC, remaining viable and preserving their membranes’ correct structure [61]. The lack of cytotoxic effect was also proven in long-term in vivo studies conducted by Pértile et al., who subcutaneously implanted BC in mice for 2 and 4 months. The post-implantation observation showed that the BC fibrils accumulated intracellularly in subcutaneous foamy macrophage aggregates. No differences between the control and implanted animals with regard to population of thymocytes, B lymphocyte precursors and myeloid cells in the bone marrow were observed [26]. In turn, Lai et al. showed the opposite type of results, i.e., the drop of fibroblast viability after 48- and 72-h exposure to BC. It is noteworthy that in this research, the chemically modified BC was examined (TEMPO (2,2,6,6-tetramethylpyperidine-1-oxyl)-mediated modified bacterial cellulose) instead of the native, cleansed polymer, and such a fact may be a reason behind the observed increased cytotoxicity [62].

To the best of the authors’ knowledge, our research presents the longest in vitro observation of fibroblast colonisation of BC-coated meshes compared to uncoated meshes (60 days). Such a long observation period allowed us to catch the moment of collapse of cell growth after the 32nd day of culture (Figure 3). Taking into consideration the small area of growth surface, such alternating changes in fibroblast numbers may by the result of filling the entire available space by cells, resulting in contact inhibition typical for a cellular monolayer. During the 60 days of the experiment, the cell culture was not passaged, so after monolayer formation, the well-proliferated fibroblasts could break the contact inhibition and create a multilayer structure.

The fibroblast quantity in samples modified with BC was higher compared to the quantity of fibroblasts in the native meshes. It may be hypothesized that the complex surface (containing fibres and pores) of BC provided fibroblasts with the additional space to attach and develop, comparing the surface of non-modified meshes. The obtained results showed that the surface of (native) mesh pores have an impact on fibroblast attachment and the growth rate. The uncoated M1 mesh pores were the largest (average area over 5.3 mm^2^) among all tested native meshes, while the fibroblast growth on this particular sample was the lowest (Table S1, Figure S8). The modification of M1 with BC increased the pace of fibroblast growth to the level comparable to that assessed for BC-M2 and BC-M3 samples. Not only the pore size, but also the native mesh material had an impact on the level of fibroblast attachment. The M2 and M3 meshes were made of polypropylene, while M1 was made of polypropylene with polyvinylpyrrolidone-polyethylene glycol additive. Covering the mesh material by BC allowed us to obtain fibroblast growth on a similar level to other two BC-coated meshes, namely M2 and M3.

Comparison of the differences in average fibroblast quantity between first and last day of observation revealed that in uncoated meshes, the disproportion was larger than in BC-coated meshes (M1: 5.8 times fibroblast quantity increase vs. 1.7 times; M2: 13.6 times vs. 3.8 times; M3: 19.3 times vs. 2.3 times, respectively) because of the fact that fibroblasts on BC-coated meshes grew more rapidly in the first days of culture than was observed in the case of uncoated meshes. In the 4th day of culture, the average fibroblast quantity on BC-coated M1 mesh was 7.1 times larger than on uncoated M1. In the case of the M2 sample, this difference was 4.3 times, and in the case of sample M3, 9.6 times. The bacterial cellulose structure allows fibroblasts to attach and overgrow BC-coated meshes to a greater extent than uncoated mesh surfaces. On BC-coated samples, the first growth peak was observed on the 12th day of culture, while on uncoated meshes, the first peak was observed on the 32nd day of culture. The absorbance measurements, reflecting the amount of living cells, were comparable between the 12th day of culture on BC-coated meshes and the 32nd day of culture on uncoated ones (BC-coated meshes on the 12th day of culture: 0.211, 0.245 and 0.220 vs. uncoated meshes on the 32nd day of culture: 0.036, 0.242 and 0.221, for M1, M2 and M3, respectively). The difference between BC-coated and uncoated meshes decreased with the duration of the culture (Table S12 and Figure S12). For M1 mesh, the differences were the most noticeable during the entire culture time. The highest one was observed on the 12th day of culture (20.9 times more fibroblasts on BC-coated than on uncoated mesh), and then there was a decrease on the 36th day (6.7 times), and on 40th day the fibroblast quantity was slightly smaller than on uncoated mesh. Between the 44th and 60th days, the difference was comparable, and it was between 2.1 and 3 times. For M2 mesh, the differences were the least noticeable during the entire culture time. The highest differences were observed on the 4th, 8th, 12th and 16th days of culture (4.3; 3.4; 2.7 and 1.9 times, respectively); after these time-points, the differences were comparable (in a spectrum between 0.7 and 1.4 times). For the M3 sample, the highest differences were observed on the 4th, 8th and 12th day of culture (9.6; 6.0 and 7.2 times, respectively), and then the differences were comparable and were between 1.1 and 1.8 times. These differences show that surgical meshes with BC coatings are covered with fibroblasts in the first days after implantation faster than uncoated meshes (which may contribute to the acceleration of the healing process in the clinical conditions). These results of our work are partially reflected in Zharikov et al.’s in vivo studies. Zharikov’s research team compared polypropylene surgical mesh to a bacterial cellulose sheet. They obtained BC from *Medusomyces gisevii* (also referred to as the “tea fungus”—a coculture of acetic bacteria and yeast). BC membrane and surgical mesh were implanted into dogs’ abdominal walls and harvested postoperatively after 14 and 60 days. After the first time-point mentioned, immature connective tissue, slight fibrinous adhesions and elements of interfacial aseptic inflammation around BC membrane were observed. After 60 days, active signs of collagen synthesis around the BC and formation of new capillary vessels were observed. Fewer intraperitoneal adhesions between the intestinal loops and the BC membrane as opposed to the polypropylene mesh were also noticed [63]. Zharikov et al. compared BC to surgical mesh after 60 days only. After 14 days they described only BC membrane, which makes it impossible for us to compare both materials in the first days after implantation. Nevertheless, the results presented after 60 days after implantation allow to conclude that BC is at least as a good material for surgical mesh as polypropylene, which is consistent with our results. In the in vivo research of Helenius et al., the BC was implanted subcutaneously in rats for 1, 4 and 12 weeks. It occurred that BC was very well integrated into the host tissue and did not elicit any chronic inflammatory reactions [64]. In Lai et al.’s research, the BC was implanted in a rabbit subcutaneous model. After one week it was demonstrated that BC mesh was fully biocompatible and integrated into surrounding tissues. A long-term (90 days) study using a ewe vaginal implantation model showed no foreign body reactions [65]. An opposite result was obtained by Ai et al., who studied BC meshes as a material for pelvic organ prolapse treatment in an in vivo sheep model. After 12 weeks post-implantation, the BC mesh resulted in less fibrosis and a higher inflammatory response than control surgical mesh [66]. Our in vitro results are consistent with other research teams’ in vitro results and also with (to the level indicated by careful extrapolation) with in vivo studies performed on such small animals as rats and rabbits. Studies with larger animals such as dogs or sheep give inconsistent results, and there is very little research of this kind using BC [63,64,65,66].

It should be noted that the porous ad fibrillar structure of BC creates an attractive environment not only for eukaryotic cells but also for a broad spectrum of microorganisms, as we showed in earlier works of our team [58,67]. Therefore, the provision of an appropriate antimicrobial to fortify the BC structure and protect it (together with mesh) from the bacterial colonization is of paramount meaning with regard to the analysed matter.

Therefore, the second aspect evaluated in our research concerned antimicrobial activity of BC-coated mesh saturated with antibacterial substance, namely gentamycin antibiotic. The rationale behind choice of this antibiotic was its successful application in implants referred to as the gentamycin (garamycin) sponges. These are foamy collagen-based sponges saturated with gentamicin sulphate of concentration 2 mg/cm^2^. When applied to a wound, the collagen breaks down, and the gentamicin is released. It was shown that the majority of gentamycin molecules remain in the wound, and only a minor part of this antibiotic is absorbed into the blood stream [68]. To saturate BC with an appropriate concentration of gentamicin, the water-holding capacity of BC was measured (Figure 6). The results showed that BC water content is about 99%, which is consistent with data presented in other research [69,70,71]. Two concentrations of gentamicin were applied in our research—4 mg/mL (the same as one applied in gentamycin sponge), and 0.47 µg/mL (equal to the MIC value against *Staphylococcus aureus* ATCC 33591 (Figure 5)). The gentamicin concentrations used displayed no harmful effects on fibroblasts, as was shown in numerous other studies [56,72,73,74]. BC-coated and uncoated meshes were saturated with both concentrations of gentamicin, and their antimicrobial activity was evaluated using a modified disc-diffusion method. For the BC-coated and uncoated meshes fortified with the higher concentration of antibiotic, the release profiles were performed. As shown in Figures S10 and S11, the BC alone released the gentamycin to the 60th minute of experiment, while this specific “plateau” time point was observed after 30 min in the cases of G-BC-M1 and -M2 and after 45 min in the case of G-BC-M3. In turn, the uncoated meshes released lower concentrations of gentamicin compared to the coated meshes, and the plateau point was reached in 5 min (for M1 and M3). In the case of M2, the gentamicin was released (to a major extent) in a time period shorter than 5 min. The above results may be explained by the fact that coated meshes displayed a greater surface area for gentamicin incorporation (thanks to the multilayer and porous structure of BC) compared to the uncoated meshes. This assumption may be additionally backed-up by the observation that BC-M3, which incorporated the highest concentration of gentamicin (compared to BC-M1 and BC-M2, Figure 1) was in its native (uncoated) form the structure of the lowest meshwork. This means that the amount of BC formed between the M3 mesh fibrils was the highest one among analysed meshes. Therefore, the amount of gentamicin that could be incorporated was higher comparing to BC-M1 and M2. In turn, data presented in Figure S11 showed very rapid release of gentamicin from uncoated meshes, suggesting rather surface than in-depth adhesion of this antibiotic to the meshes’ fibrils.

Uncoated meshes saturated with 0.47 µg/mL of gentamicin did not inhibit bacterial growth. Among BC-coated samples, 50% inhibited bacterial growth (growth inhibition zones between 11 and 20 mm). Higher gentamicin concentration was effective both in BC-coated and uncoated samples. Growth inhibition zones were slightly larger in BC-coated samples (Figure 7). Our results showed that meshes with a BC layer can absorb and release more liquid antibacterial substance than uncoated meshes on their fibres. Thanks to the very high water capacity of BC and its nano-filamentary structure, BC can absorb enough fluid to maintain the antimicrobial effect. Various BC modifications were tested in order to fortify BC with antibacterial properties [75,76,77]. In our previous research, we demonstrated antibiofilm activity of wound dressing made of BC and saturated with different antiseptics. Results proved that BC could absorb and release also these other antimicrobials to an extent high enough to eradicate bacteria [78]. To the best of the authors’ knowledge, despite of a lot of research describing modified BC dressings for infected wounds, only one study was performed to evaluate antimicrobial-modified BC in a character of hernia mesh. Liu et al. synthetised the cellulose/collagen–hydroxypropyltrimethyl ammonium chloride chitosan composite (BCC-H). Their composites’ biocompatibility was slightly lower but comparable to native BC. The bacteriostatic rates of BCC-1.0H and BCC-0.5H reached up to 99% and 88%, respectively [79].

The BC-coated surgical meshes, analysed in the current research, displayed no cytotoxic effect and high biocompatibility toward fibroblast in vitro culture. In addition, the pace of fibroblast growth was faster in BC-coated meshes compared to the native meshes in the first days of culturing. The nanofibrous structure of BC and its high water capacity allowed BC to be saturated with antimicrobial gentamycin, able to eradicate *Staphylococcus aureus* pathogen. The results of our study, although requiring thorough verification in a clinical setting, indicate that BC-modified hernia meshes, fortified with an appropriate antimicrobial, may be applied as effective implants in hernia surgery, preventing risk of infection occurrence.

## 4. Materials and Methods

**Figure 8:**
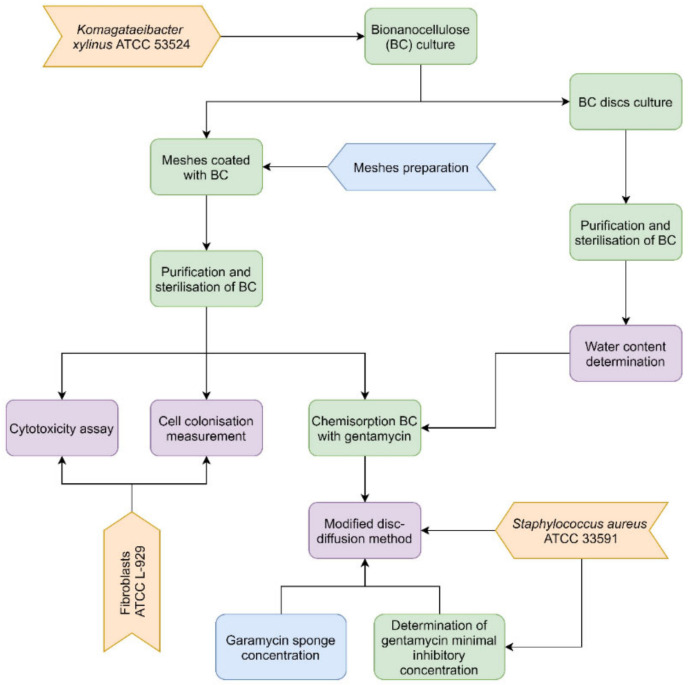
The research scheme.

The research scheme is presented in Figure 8.

### 4.1. Sample Preparation

As a base, three commercially-available surgical meshes were used:

- non-absorbable mesh made from polypropylene-polyvinylpyrrolidone-polyethylene glycol with adhesive layer (Adhesix™, BARD, New Providence, NJ, USA), later referred to as the M1.
- non-absorbable macroporous mesh made from polypropylene microfilaments (Hermesh 4, Polhernia, Gdansk, Poland), later referred to as the M2.
- non-absorbable macroporous mesh made from polypropylene monofilaments, knitted with quadriaxial technology (Hermesh 8, Herniamesh^®^ S.r.l. Chivasso, Turin, Italy), later referred to as the M3.

The mesh pore sizes were measured using the OmniDOC Gel Documentation System (Cleaver Scientific, Rugby, Warwickshire, UK). Meshes were cut to rectangles with dimensions 8 × 9 mm, sterilized in a steam autoclave (Vapour Line, VWR, Radnor, PA, USA) and placed into sterile 24-well plates (VWR, Radnor, PA, USA). An adhesive layer of M1 was destroyed during the sterilization process. Then, 0.3 mL *of Komagataeibacter xylinus* ATCC 53524 (American Type Culture Collection, Manassas, VA, USA) suspension with 0.5 McF density (McFarland scale; DensiLaMeter II, Erba Lachema, Brno, Czech Republic) was added into each well, and plates were incubated at 28 °C in aerobic conditions. Every day, fresh Hestrin–Schramm culture medium (HS; 2% glucose (*w*/*v*; Chempur, Piekary Slaskie, Poland), 0.5% yeast extract (*w*/*v*; VWR Chemicals, Radnor, PA, USA), 0.5% bactopeptone (*w*/*v*; VWR Chemicals, Radnor, PA, USA), 0.115% citric acid (*w*/*v*; POCH, Gliwice, Poland), 0.27% Na_2_HPO_4_ (*w*/*v*; POCH, Gliwice, Poland), 0.05% MgSO_4_ heptahydrate (*w*/*v*; POCH, Gliwice, Poland) and 1% ethanol (*v*/*v*; Stanlab, Lublin, Poland)) were added. After 3 days of incubation, samples were turned upside down for uniform coverage of cellulose. Samples were incubated 3 days more, and fresh HS medium was added every day. Covered meshes were placed into 0.1 M NaOH solution (POCH, Gliwice, Poland) and incubated for 2 days at 80 °C to destroy bacterial cells. After chemical purification, samples were rinsed with water to obtain pH = 7 (pH strips, Macherey–Nagel, Düren, Germany) and sterilized in a steam autoclave (Vapour Line, VWR, Radnor, PA, USA).

### 4.2. Cytotoxicity Assay

A neutral red cytotoxicity assay was performed in fibroblast ATCC CCL-1 cell cultures. The procedure was prepared according to the ISO 10993 norm: Biological evaluation of medical devices; Part 5: Tests for in vitro cytotoxicity; and Part 12: Biological evaluation of medical devices, sample preparation and reference materials (ISO 10993-5:2009 and ISO/IEC 17025:2005) [80].

BC covered with meshes were placed into 5.0 mL Eppendorf tubes (Eppendorf, Hamburg, Germany), and 2.0 mL of high glucose DMEM medium (Biowest, Nuaillé, France) was added. Samples were incubated for 24 h and 48 h in 5% CO_2_ at 37 °C. After incubation, meshes were taken out from the tubes, and supernatants were used for further research. Fibroblast cell line ATCC CCL-1 was cultivated in high glucose DMEM (Biowest, Nuaillé, France) with the addition of 1% amphotericin B (Biowest, Nuaillé, France), 1% penicillin (Biowest, Nuaillé, France) and 10% bovine serum (Biowest, Nuaillé, France) in 5% CO_2_ at 37 °C. To mature fibroblasts, trypsin-EDTA 1X (Biowest, Nuaillé, France) was added to remove cells from culture flasks (Sarstedt, Nümbrecht, Germany). The cell suspension with a density of 10^5^ cells/mL was prepared and added into the wells of 96-well plates (VWR, Radnor, PA, USA) in volumes of 100 µL per well. Cells were incubated for 24 h in 5% CO_2_ at 37 °C, medium was removed and previously prepared supernatants were added to cells (100 µL each well). As a control sample, supernatants from non-coated meshes were used, and high glucose DMEM medium (Biowest, Nuaillé, France) instead of supernatants was added as a cell growth control. Samples were incubated for 24 h in 5% CO_2_ at 37 °C. After incubation, supernatants were removed, 100 µL of neutral red solution (NR, Merck, Darmstadt, Germany) was added into wells and samples were incubated for 3 h in 5% CO_2_ at 37 °C. NR solution was removed, and 100 µL of an extraction mixture (50% ethyl alcohol (POCH, Gliwice, Poland), 49% water and 1% glacial acetic acid (POCH, Gliwice, Poland), *v*/*v*) was added. Plates were shaken for 15 min at 400 rpm (Thermo Shaker PST-60HL-4, Biosan, Riga, Latvia), and the value of NR absorbance was measured spectrophotometrically (Multi-scan GO, Thermo Fisher Scientific, Waltham, MA, USA) at 540 nm wavelength.

### 4.3. Cell Colonisation Measurement

To perform the cell colonisation assay, fibroblast ATCC CCL-1 cell cultures were used. Uncoated and BC-coated meshes were placed in wells of 24-well plates (VWR, Radnor, PA, USA), and into each well 2.0 mL of fibroblast suspension in high glucose DMEM culture medium (Biowest, Nuaillé, France) with serum (10%, *v*/*v*, Biowest, Nuaillé, France) and antibiotics (1% penicillin, 1% amphotericin, *v*/*v*, Biowest, Nuaillé, France) was added (density of 10^5^ cells/mL). Samples were incubated for 60 days in 5% CO_2_ at 37 °C. Every two days, culture medium was changed to fresh, and every four days, cell viability was measured using neutral red dye (staining method similar as in the cytotoxicity assay, described in Section 2.2). On the 4th, 16th, 28th, 40th, 52nd and 60th day of culture, samples were visualised under an inverted microscope (Olympus CKX41, Olympus, Shinjuku, Tokyo, Japan) and scanning electron microscope (SEM, Zeiss EVO MA25, Oberkochen, Germany). Before measurement of fibroblast quantity, samples were transferred to the fresh, new plates so cells attached to plates did not contribute to the measurement of cellular viability performed for cells attached to cellulose or meshes.

### 4.4. Determination of Minimal Inhibitory Concentration (MIC) and Minimal Biofilm Eradication Concentration (MBEC) of Gentamicin

The microdilution method was used to determine the MIC value of gentamicin (Oxoid, Thermo Fisher Scientific, Hampshire, UK). Reference strain *Staphylococcus aureus* ATCC 33591 was cultured in tryptic-soy broth (TSB, Biomaxima, Lublin, Poland) for 24 h at 37 °C in aerobic conditions. Bacterial suspension density was measured using a densitometer (DensiLaMeter II, Erba Lachema, Brno, Czech Republic) and diluted to 1.5 × 10^5^ cells/mL. In 96-well plates (VWR, Radnor, PA, USA) dilutions of gentamicin in TSB were made in a volume 100 µL (tested concentration range was from 1.55 mg/mL to 0.03 µg/mL), and 100 µL of bacterial suspension was added. Plates were incubated for 24 h at 37 °C with shaking at 400 rpm (Thermo Shaker PST-60HL-4, Biosan, Riga, Latvia). After incubation, 20 µL of 1% solution of 2,3,5-triphenyltetrazolium chloride (TTC, PanReac AppliChem, Darmstadt, Germany) in TSB was added, and samples were incubated for 2 h more at 37 °C with shaking at 400 rpm. Before and after the first incubation, spectrophotometrically measurements were taken (λ = 580 nm, Multiscan Go, Thermo Fisher Scientific, Waltham, MA, USA). After incubation with TTC, the results were read based on culture colour change to red.

To evaluate the MBEC value of gentamicin (Oxoid, Thermo Fisher Scientific, Hampshire, UK), reference strain *Staphylococcus aureus* ATCC 33591 was cultured in TSB (Biomaxima, Lublin, Poland) for 24 h at 37 °C in aerobic conditions. Bacterial suspension density was measured using a densitometer (DensiLaMeter II, Erba Lachema, Brno, Czech Republic) and diluted to 1.5 × 10^5^ cells/mL. Then, 200 µL of diluted bacterial suspension was placed in 96-well plates (VWR, Radnor, PA, USA) and incubated for 24 h at 37 °C in static and aerobic conditions. After incubation, culture medium was removed over the formed biofilms, and dilutions of gentamicin in TSB in a volume 100 µL and 100 µL of fresh TSB were added (tested concentration range the same as in MIC test) Plates were incubated for 24 h at 37 °C in static conditions. After incubation, culture medium was removed over the biofilms, and 200 µL of 0.1% solution of TTC (PanReac AppliChem, Darmstadt, Germany) in TSB was added. Samples were incubated for 2 h more at 37 °C. TTC was removed, and 100 µL of methanol was added. Plates were incubated 20 min at 37 °C with shaking at 400 rpm. Spectrophotometrically measurements were taken (λ = 490 nm, Multiscan Go, Thermo Fisher Scientific, Waltham, MA, USA).

### 4.5. Bacterial Cellulose Water Content Determination

To determine water content in bacterial cellulose, a disc from BC was prepared. First, 100 µL of 0.5 McF density (DensiLaMeter II, Erba Lachema, Brno, Czech Republic) of *Komagataeibacter xylinus* ATCC 53524 suspension was added to 2.0 mL of Hestrin–Schramm medium (self-prepared based on composition described in Section 2.1) and incubated for 7 days at 28 °C. Discs were removed from plates and purified using 0.1 M NaOH solution (POCH, Gliwice, Poland) at 80 °C for 3 days with daily changes of NaOH. After chemical purification, samples were rinsed with water to obtain pH = 7 (pH strips, Macherey–Nagel, Düren, Germany), sterilized in a steam autoclave (Vapour Line, VWR, Radnor, PA, USA) and weighed (Pioneer PA 114CM/1, OHAUS, Parsippany, NJ, USA). BC discs were dried for 24 h at 37 °C and weighed again. Furthermore, BC-coated and uncoated meshes were weighed.

### 4.6. Chemisorption of BC-Coated Meshes with Gentamicin and Determination of Its Release Profile

To determine the appropriate concentration of gentamicin needed to saturate BC-coated meshes, the amount of BC on meshes had to be specified. For this purpose, pieces of meshes were aseptically weighed (Pioneer PA 114CM/1, OHAUS, Parsippany, NJ, USA) before coating with BC and after coating, purification and sterilization processes. Samples were prepared with concentrations of MIC (determined during current research) and 4.0 mg/mL of gentamicin (Oxoid, Thermo Fisher Scientific, Hampshire, UK) in BC-coated and uncoated meshes. Samples were placed in 24-well plates (VWR, Radnor, PA, USA), and 0.5 mL of gentamicin solutions was added. Incubation lasted for 24 h at 4 °C. As a negative control (no antimicrobial effect), samples were saturated with 0.9% of NaCl (Stanlab, Lublin, Poland). The release profile of gentamicin (4 mg/mL) from uncoated and coated meshes was determined analogically to the procedures performed in our earlier publication [57].

### 4.7. Modified Disc Diffusion Method

*S. aureus* ATCC 33591 strain was cultured in TSB medium (Biomaxima, Lublin, Poland) at 37 °C in aerobic conditions for 24 h and then diluted to 0.5 McF density (DensiLaMeter II, Erba Lachema, Brno, Czech Republic). Bacterial suspension was cultured on a Petri dish with Mueller–Hinton Agar (Biomaxima, Lublin, Poland). The saturated BC-coated and uncoated meshes were placed in the middle of the dish and incubated upside down for 24 h at 37 °C in aerobic conditions. After incubation time, the growth inhibition zones were measured by a ruler along the diagonal of the samples.

### 4.8. Statistical Evaluation

Statistical analyses were performed using GraphPad Prism 8.0.1 and 9.3.1 (GraphPad Software, San Diego, CA, USA). Descriptive statistics included arithmetic mean, standard deviation, standard error of the mean, median and coefficient of variation. Whiskers on graphs showed median with 95% of confidence interval. Normality of distribution was verified using Shapiro–Wilk’s test. To evaluate statistical significance Kruskal–Wallis multiple comparisons test with post hoc Dunne’s modification (α = 0.05) or one-way ANOVA test with post hoc Tukey modification (α = 0.05) were performed.

## Data Availability

All data are provided in the main body of the manuscript and Supplementary Materials.

## Supplementary Materials

The following supporting information can be downloaded at: https://www.mdpi.com/article/10.3390/ijms23094835/s1.
