# Supplementary material for: In Vitro Cytotoxicity, Colonisation by Fibroblasts and Antimicrobial Properties of Surgical Meshes Coated with Bacterial Cellulose
Source: Int J Mol Sci. 2022 Apr 27;23(9):4835. doi: 10.3390/ijms23094835 (PMC9105287; doi:10.3390/ijms23094835)
Supplement: Supplementary file 1 [file ijms-23-04835-s001.zip › ijms-1679076-supplementary.pdf]

International Journal of *Molecular Sciences*

MDPI

Supplementary Materials

In Vitro Cytotoxicity, Colonization by Fibroblasts and Antimicrobial Properties of Surgical Meshes Coated With Bacterial Cellulose

Karolina Dydak[1,*], Adam Junka[1,*], Grzegorz Nowacki[2], Justyna Paleczny[1], Patrycja Szymczyk-Ziółkowska[3], Aleksandra Górzyńska[4], Olga Aniołek[5], Marzenna Bartoszewicz[1]

1 Department of Pharmaceutical Microbiology and Parasitology, Wroclaw Medical University, Borowska 211a, 50-556 Wroclaw, Poland; karolina.dydak@umw.edu.pl; adam.junka@umw.edu.pl; justyna.paleczny@student.umw.edu.pl; marzenna.bartoszewicz@umw.edu.pl
2 Department of Laboratory Diagnostics Health Care Complex in Nysa, Bohaterów Warszawy 23, 48-300 Nysa, Poland; grzesiek.nowacki95@gmail.com
3 Centre for Advanced Manufacturing Technologies (CAMT/FPC), Faculty of Mechanical Engineering, Wroclaw University of Science and Technology, Łukasiewicza 5, 50-371Wroclaw, Poland, patrycja.e.szymczyk@pwr.edu.pl
4 Student Research Circle at the Department of Pharmaceutical Microbiology and Parasitology, Wroclaw Medical University, Borowska 211a, 50-556 Wroclaw, Poland; ola.gorzynska@wp.pl
5 Faculty of Medicine, Lazarski University, 02-662 Warsaw, Poland; olga.aniolek@lazarski.pl
* Correspondence:
karolina.dydak@umw.edu.pl; +48505563559; Borowska 211a, 50-556 Wroclaw, Poland
adam.junka@umw.edu.pl; +48889 229 341; Borowska 211a, 50-556 Wroclaw, Poland

Citation: Dydak, K.; Junka, A.; Nowacki, G.; Paleczny, J.; Szymczyk-Ziółkowska, P.; Górzyńska, A.; Aniołek, O.; Bartoszewicz, M. In Vitro Cytotoxicity, Colonization by Fibroblasts and Antimicrobial Properties of Surgical Meshes Coated with Bacterial Cellulose. *Int. J. Mol. Sci.* **2022**, *23*, 4835. https://doi.org/10.3390/ijms23094835

Academic Editor: Andreas Burkovski

Received: 28 March 2022
Accepted: 25 April 2022
Published: 27 April 2022

Publisher's Note: MDPI stays neutral with regard to jurisdictional claims in published maps and institutional affiliations.

Copyright: © 2022 by the authors. Licensee MDPI, Basel, Switzerland. This article is an open access article distributed under the terms and conditions of the Creative Commons Attribution (CC BY) license (https://creativecommons.org/licenses/by/4.0/).

Abstract: Hernia repairs are the most common abdominal wall elective procedures performed by general surgeons. The hernia-related postoperative infective complications occur with 10% frequency. To counter-act the risk of infection emergence, the development of effective, biocompatible and antimicrobial mesh adjuvants is required. Therefore, the aim of our *in vitro* investigation was to evaluate the suitability of bacterial cellulose (BC) polymer, coupled with gentamicin (GM) antibiotic as an absorbent layer of surgical mesh. Our research included the assessment of GM-BC-modified meshes' cytotoxicity against fibroblasts ATCC CCL-1 and a 60 days-lasting cell colonisation measurement. Obtained results showed no cytotoxic effect of modified meshes. The quantified fibroblast cells levels resembled of bimodal distribution of specifics depended from the time of culturing and the type of mesh applied. The measured GM minimal inhibitory concentration was 0,47 µg/ml. Results obtained in modified disc-diffusion method shows that GM-BC-modified meshes inhibited bacterial growth more effectively than non-coated meshes. The results of our study indicate that BC-modified hernia meshes, fortified with appropriate antimicrobial, may be applied as effective implants in hernia surgery, preventing from risk of infection occurrence and provide high level of biocompatibility with regard to fibroblast cells.

Keywords: hernia mesh, bacterial cellulose, gentamicin, biocompatibility

Table S1. Pore surface area of meshes: M1 – Adhesix™, BARD, New Providence, New Jersey, USA, M2 – Hermesh 4, Polhernia, Gdansk, Poland, M3 – Hermesh 8, Herniamesh® S.r.l. Chivasso, Italy. AM – arithmetic mean, SD – standard deviation, SEM – standard error of the mean. Surface area of pores were determined using OmniDOC Gel Documentation System (Cleaver Scientific, Rugby, Warwickshire, United Kingdom).

Mesh	M1	M2	M3
Pore surface area [mm^2]	5.6	3.7	2.2
	6.1	3.3	1.7
	4.9	4.6	2.7
	5.0	4.2	2.1
	5.2	3.9	2.4
	5.1	3.8	1.6
**AM**	5.32	3.92	2.12
**SD**	0.45	0.44	0.42
**SEM**	0.19	0.18	0.17

Table S2. Statistical differences between tested meshes pore's surface area. M1 – Adhesix™, BARD, New Providence, New Jersey, USA, M2 – Hermesh 4, Polhernia, Gdansk, Poland, M3 – Hermesh 8, Herniamesh® S.r.l. Chivasso, Italy; Sign. Diff. – significant difference; Lv of diff. – level of difference; *** - high statistically significance; **** - very high statistically significance.

One-way ANOVA test with post-hoc Tukey's modification; $\alpha = 0.05$			
**Samples**	**Sign. Diff.**	**Lv of diff.**	**Adjusted P**
M1 vs. M2	Yes	***	0.0002
M1 vs. M3	Yes	****	< 0.0001
M2 vs. M3	Yes	****	< 0.0001

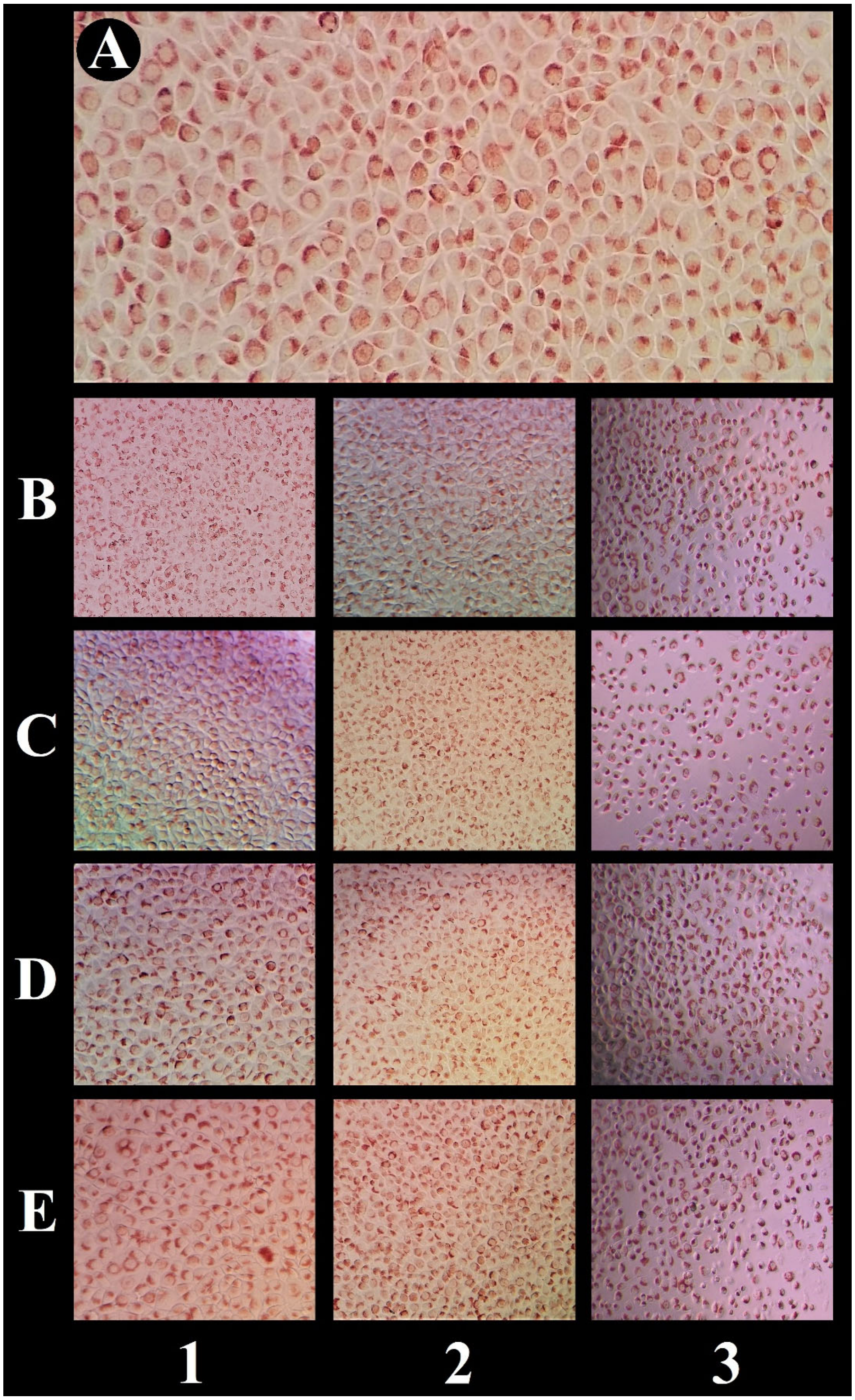

Figure S1. Pictures of fibroblast cell line ATCC CCL-1 after exposition to extracts from bacterial cellulose coated and uncoated surgical meshes. Neutral red staining. A – control sample; B – 24 h extracts from uncoated meshes; C – 24 h extract from bacterial cellulose coated meshes; D – 48 h extracts from uncoated meshes; E – 48 h extract from bacterial cellulose coated meshes; 1 – M1 mesh (Adhesix™, BARD, New Providence, New Jersey, USA); 2 – M2 mesh (Hermesh 4, Polhernia, Gdansk, Poland); 3 – M3 mesh (Hermesh 8, Herniamesh® S.r.l. Chivasso, Italy). Olympus CKX41 (Olympus, Shinjuku, Tokyo, Japan).

Table S3. Statistical data to „Cytotoxicity Assay" section. M1 (Adhesix™, BARD, New Providence, New Jersey, USA); M2 (Hermesh 4, Polhernia, Gdansk, Poland); M3 (Hermesh 8, Herniamesh® S.r.l. Chivasso, Italy); BC-M1/M2/M3 – described meshes coated with bacterial cellulose; Control – fibroblasts ATCC CCL-1 incubated with fresh culture medium; Sign. Diff. – significant difference; Lv of diff. – level of difference, ns – no significant differences; ** - moderate statistically significance.

	Kruskal-Wallis test with post-hoc Dunne's modification; $\alpha = 0.05$					
	**Sample**	**vs.**	**Sample**	**Sign. Diff.**	**Lv of diff.**	**Adjusted P**
**24 h extracts**	M1	vs.	M2	No	ns	> 0.9999
	M1	vs.	M3	No	ns	> 0.9999
	M2	vs.	M3	No	ns	> 0.9999
	M1	vs.	BC-M1	No	ns	> 0.9999
	M2	vs.	BC-M2	No	ns	> 0.9999
	M3	vs.	BC-M3	No	ns	> 0.9999
	BC-M1	vs.	BC-M2	No	ns	> 0.9999
	BC-M1	vs.	BC-M3	No	ns	> 0.9999
	BC-M2	vs.	BC-M3	No	ns	> 0.9999
	M1	vs.	Control	No	ns	> 0.9999
	M2	vs.	Control	No	ns	> 0.9999
	M3	vs.	Control	No	ns	0.3440
	BC-M1	vs.	Control	No	ns	0.2709
	BC-M2	vs.	Control	No	ns	> 0.9999
	BC-M3	vs.	Control	No	ns	> 0.9999
**48 h extracts**	M1	vs.	M2	No	ns	> 0.9999
	M1	vs.	M3	No	ns	> 0.9999
	M2	vs.	M3	No	ns	> 0.9999
	M1	vs.	BC-M1	No	ns	> 0.9999
	M2	vs.	BC-M2	No	ns	> 0.9999
	M3	vs.	BC-M3	No	ns	> 0.9999
	BC-M1	vs.	BC-M2	No	ns	> 0.9999
	BC-M1	vs.	BC-M3	No	ns	0.1945
	BC-M2	vs.	BC-M3	No	ns	> 0.9999
	M1	vs.	Control	No	ns	0.1339
	M2	vs.	Control	No	ns	0.0751
	M3	vs.	Control	No	ns	> 0.9999
	BC-M1	vs.	Control	No	ns	> 0.9999
	BC-M2	vs.	Control	No	ns	0.1263
	BC-M3	vs.	Control	Yes	**	0.0059

Table S4. Statistical comparisons of fibroblasts ATCC CCL-1 colonisation of bacterial cellulose coated and uncoated surgical meshes between 4th and 60th day of culture. M1 (Adhesix™, BARD, New Providence, New Jersey, USA); M2 (Hermesh 4, Polhernia, Gdansk, Poland); M3 (Hermesh 8, Herniamesh® S.r.l. Chivasso, Italy); BC-M1/M2/M3 – described meshes coated with bacterial cellulose; Sign. Diff. – significant difference; Lv of diff. – level of difference; ns – no significant differences; ** - moderate statistically significance; *** - high statistically significance.

Kruskal-Wallis test with post-hoc Dunne's modification; $\alpha = 0.05$					
Sample 4th day of culture	vs.	Sample 60th day of culture	Sign. Diff.	Lv of diff.	Adjusted P
M1	vs.	M1	Yes	**	0.0072
BC-M1	vs.	BC-M1	No	ns	0.0951
M2	vs.	M2	Yes	****	< 0.0001
BC-M2	vs.	BC-M2	Yes	****	< 0.0001
M3	vs.	M3	Yes	****	< 0.0001
BC-M3	vs.	BC-M3	Yes	**	0.0030

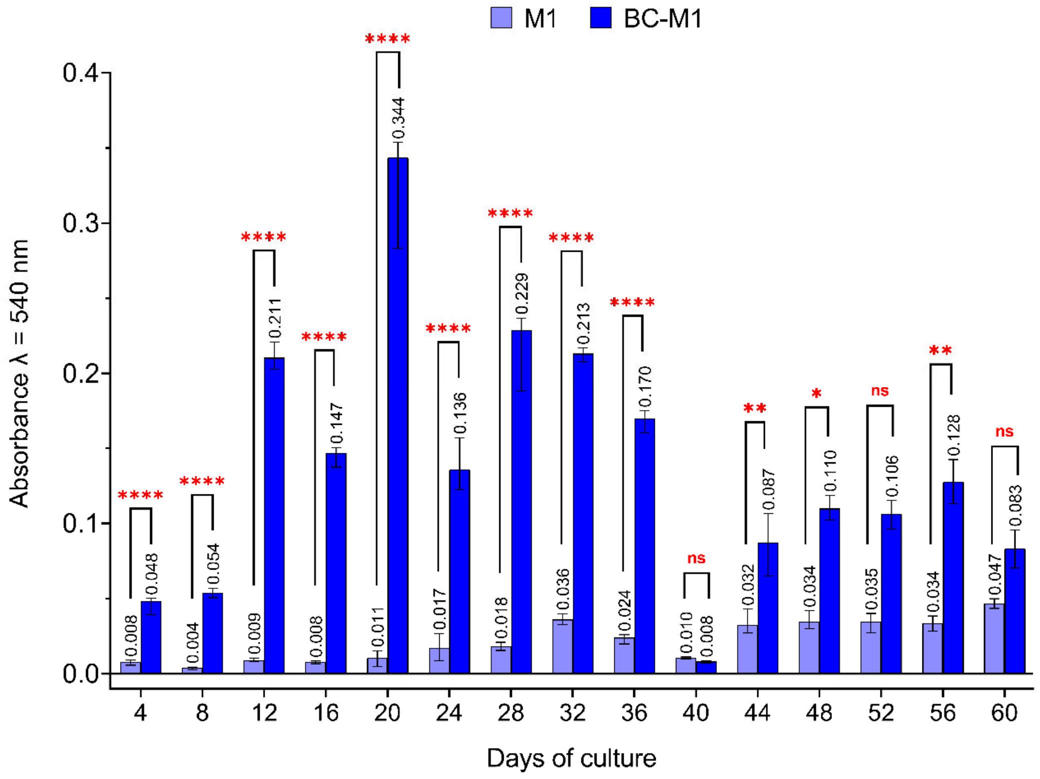

Figure S2. Fibroblasts ATCC CCL-1 colonisation of bacterial cellulose coated and uncoated surgical mesh M1. M1 (Adhesix™, BARD, New Providence, New Jersey, USA); BC-M1 – mesh M1 coated with bacterial cellulose; ns – no significant differences; * – low statistically significance (p = 0.0130); ** – moderate statistically significance (p < 0.0095); **** – very high statistically significance (p <

0.0001); ns – no significant differences; whiskers show median with 95% of confidence interval. Full statistical details are shown in Table S5.

Table S5. Statistical comparisons of fibroblasts ATCC CCL-1 colonisation between bacterial cellulose coated and uncoated surgical mesh M1 during culture period. M1 (Adhesix™, BARD, New Providence, New Jersey, USA); BC-M1 – mesh M1 coated with bacterial cellulose; Sign. Diff. – significant difference; Lv of diff. – level of difference; ns – no significant differences; * - low statistically significance, ** - moderate statistically significance; **** - very high statistically significance.

Kruskal-Wallis test with post-hoc Dunne's modification; α = 0.05				
**Day of culture**	**sample vs. sample**	**Sign. Diff.**	**Lv of diff.**	**Adjusted P**
**4**	M1 vs. BC-M1	Yes	****	< 0.0001
**8**		Yes	****	< 0.0001
**12**		Yes	****	< 0.0001
**16**		Yes	****	< 0.0001
**20**		Yes	****	< 0.0001
**24**		Yes	****	< 0.0001
**28**		Yes	****	< 0.0001
**32**		Yes	****	< 0.0001
**36**		Yes	****	< 0.0001
**40**		No	ns	> 0.9999
**44**		Yes	**	0.0095
**48**		Yes	*	0.0130
**52**		No	ns	0.0681
**56**		Yes	**	0.0091
**60**		No	ns	0.1964

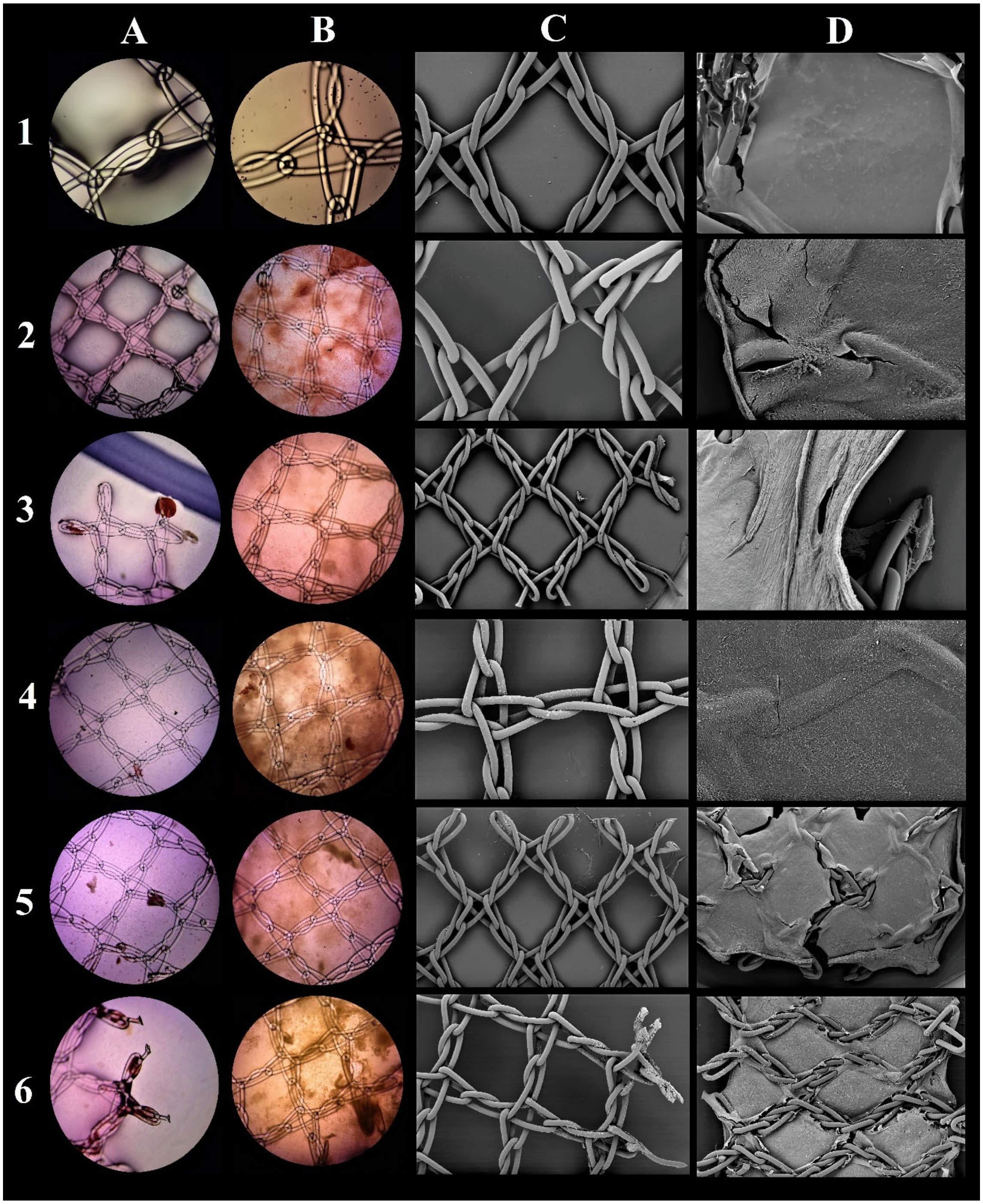

Figure S3. Visualisation of fibroblasts ATCC CCL-1 on bacterial cellulose coated and uncoated surgical mesh M1. M1 (Adhesix™, BARD, New Providence, New Jersey, USA); A, B – neutral red staining, light microscope (Olympus CX23, Shinjuku, Tokyo, Japan); C, D – scanning electron microscope (Zeiss EVO MA25, Oberkochen, Germany); A, C – uncoated meshes; B, D – bacterial cellulose coated meshes; 1–6 – 4th, 16th, 28th, 40th, 52nd and 60th day of culture, respectively.

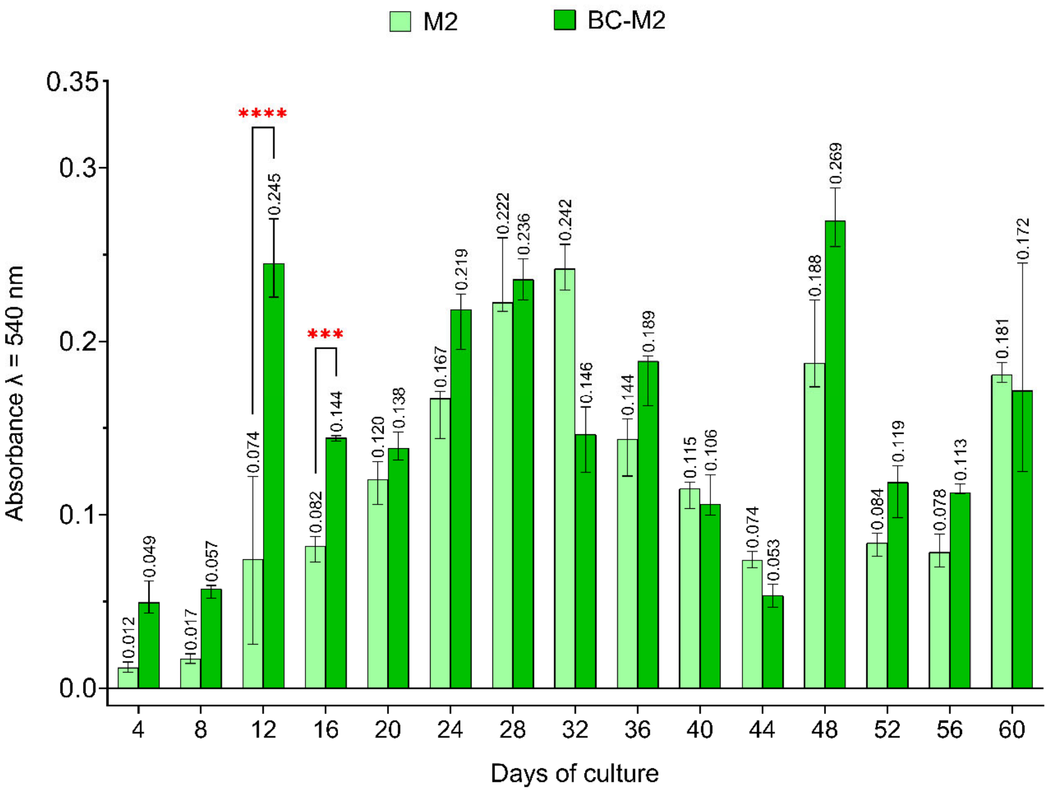

Figure S4. Fibroblasts ATCC CCL-1 colonisation of bacterial cellulose coated and uncoated surgical mesh M2. M2 (Hermesh 4, Polhernia, Gdansk, Poland); BC-M2 – mesh M2 coated with bacterial cellulose; *** – high statistically significance ($p = 0.0002$); **** – very high statistically significance ($p < 0.0001$); whiskers show median with 95% of confidence interval. Full statistical details are shown in Table S6.

Table S6. Statistical comparisons of fibroblasts ATCC CCL-1 colonisation between bacterial cellulose coated and uncoated surgical mesh M2 during culture period. M2 (Hermesh 4, Polhernia, Gdansk, Poland); BC-M2 – mesh M2 coated with bacterial cellulose; Sign. Diff. – significant difference; Lv of diff. – level of difference; ns – no significant differences; *** - high statistically significance; **** - very high statistically significance.

Kruskal-Wallis test with post-hoc Dunne's modification; $\alpha = 0.05$				
**Day of culture**	**sample vs. sample**	**Sign. Diff.**	**Lv of diff.**	**Adjusted P**
**4**	M2 vs. BC-M2	No	ns	> 0.9999
**8**		No	ns	> 0.9999
**12**		Yes	****	< 0.0001
**16**		Yes	***	0.0002
**20**		No	ns	> 0.9999
**24**		No	ns	> 0.9999
**28**		No	ns	> 0.9999
**32**		No	ns	0.0834
**36**		No	ns	> 0.9999
**40**		No	ns	> 0.9999
**44**		No	ns	> 0.9999
**48**		No	ns	0.2499
**52**		No	ns	0.5708
**56**		No	ns	0.5523
**60**		No	ns	> 0.9999

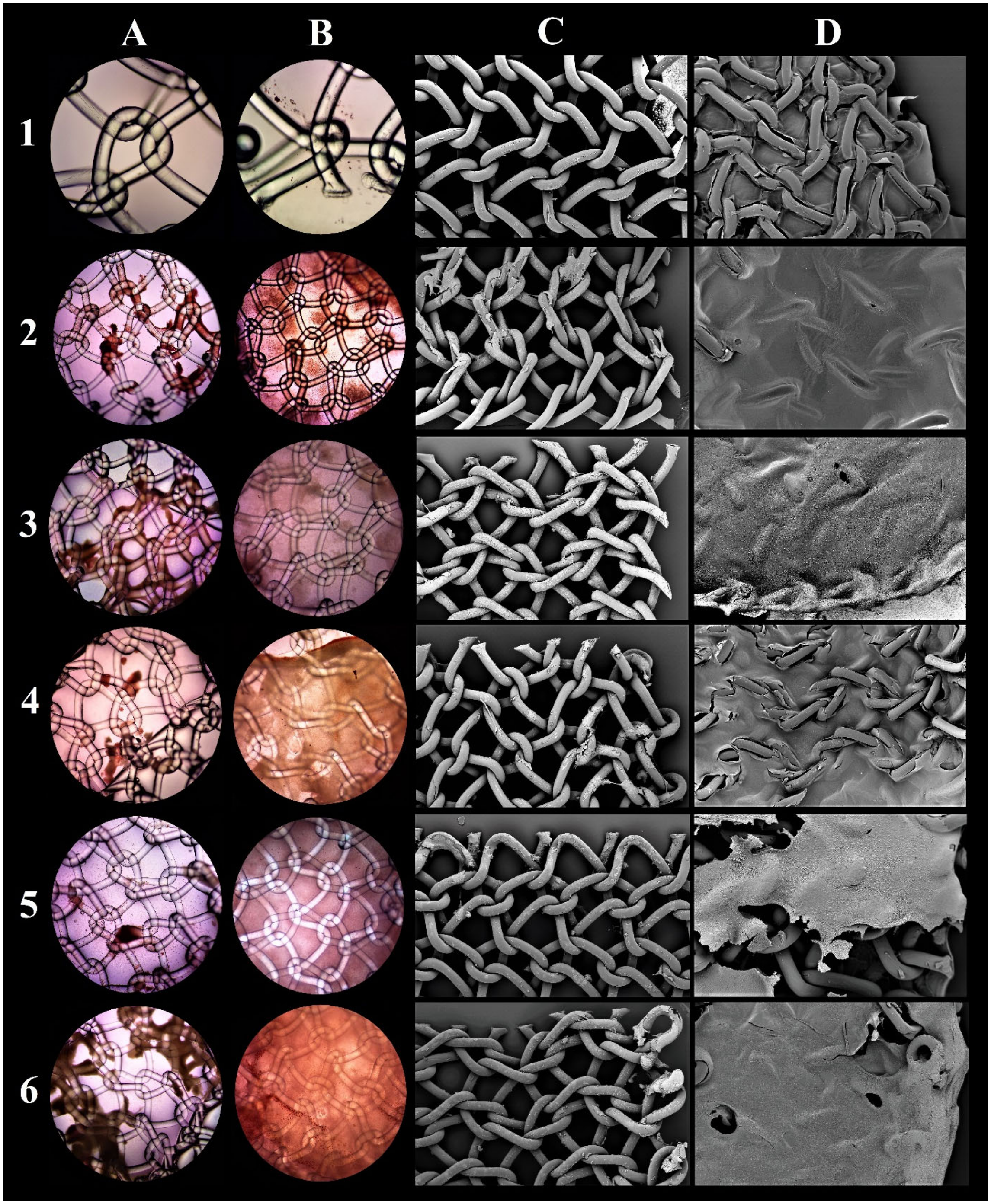

Figure S5. Visualisation of fibroblasts ATCC CCL-1 on bacterial cellulose coated and uncoated surgical mesh M2. M2 (Hermesh 4, Polhernia, Gdansk, Poland); A, B – neutral red staining, light microscope (Olympus CX23, Shinjuku, Tokyo, Japan); C, D – scanning electron microscope (Zeiss EVO MA25, Oberkochen, Germany); A, C – uncoated meshes; B, D – bacterial cellulose coated meshes; 1–6 – 4th, 16th, 28th, 40th, 52nd and 60th day of culture, respectively.

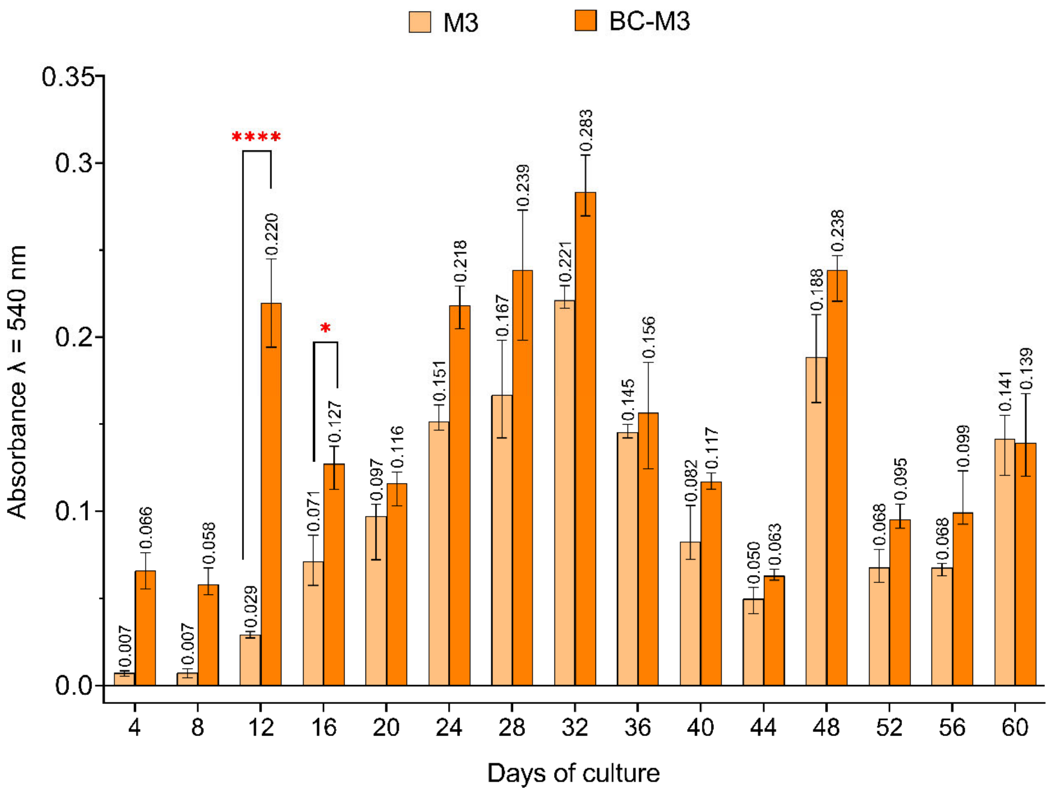

Figure S6. Fibroblasts ATCC CCL-1 colonisation of bacterial cellulose coated and uncoated surgical mesh M3. M3 (Hermesh 8, Herniamesh® S.r.l. Chivasso, Italy); BC-M3 – mesh M3 coated with bacterial cellulose; * – low statistically significance ($p = 0.0108$); **** – very high statistically significance ($p < 0.0001$); whiskers show median with 95% of confidence interval. Full statistical details are shown in Table S7.

Table S7. Statistical comparisons of fibroblasts ATCC CCL-1 colonisation between bacterial cellulose coated and uncoated surgical mesh M3 during culture period. M3 (Hermesh 8, Herniamesh® S.r.l. Chivasso, Italy); BC-M3 – mesh M3 coated with bacterial cellulose; Sign. Diff. – significant difference; Lv of diff. – level of difference; ns – no significant differences; * low statistically significance; **** - very high statistically significance.

Kruskal-Wallis test with post-hoc Dunne's modification; α = 0.05				
**Day of culture**	**sample vs. sample**	**Sign. Diff.**	**Lv of diff.**	**Adjusted P**
**4**	M3 vs. BC-M3	No	ns	0.1409
**8**		No	ns	0.3574
**12**		Yes	****	< 0.0001
**16**		Yes	*	0.0108
**20**		No	ns	0.9864
**24**		No	ns	0.4847
**28**		No	ns	> 0.9999
**32**		No	ns	> 0.9999
**36**		No	ns	> 0.9999
**40**		No	ns	0.7210
**44**		No	ns	> 0.9999
**48**		No	ns	> 0.9999
**52**		No	ns	> 0.9999
**56**		No	ns	0.8566
**60**		No	ns	> 0.9999

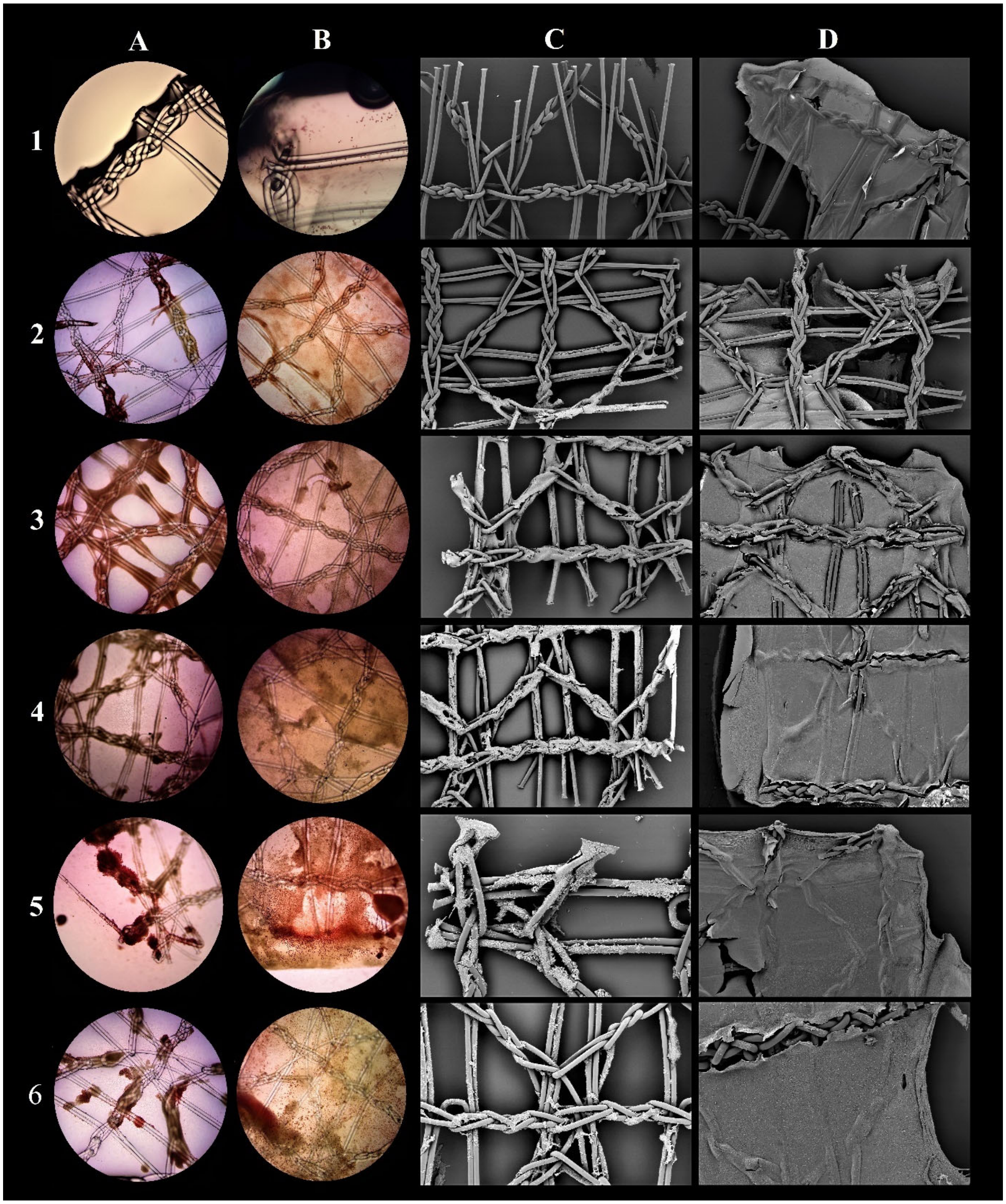

Figure S7. Visualisation of fibroblasts ATCC CCL-1 on bacterial cellulose coated and uncoated surgical mesh M3. M3 (Hermesh 8, Herniamesh® S.r.l. Chivasso, Italy); A, B – neutral red staining, light microscope (Olympus CX23, Shinjuku, Tokyo, Japan); C, D – scanning electron microscope (Zeiss EVO MA25, Oberkochen, Germany); A, C – uncoated meshes; B, D – bacterial cellulose coated meshes; 1–6 – 4th, 16th, 28th, 40th, 52nd and 60th day of culture, respectively.

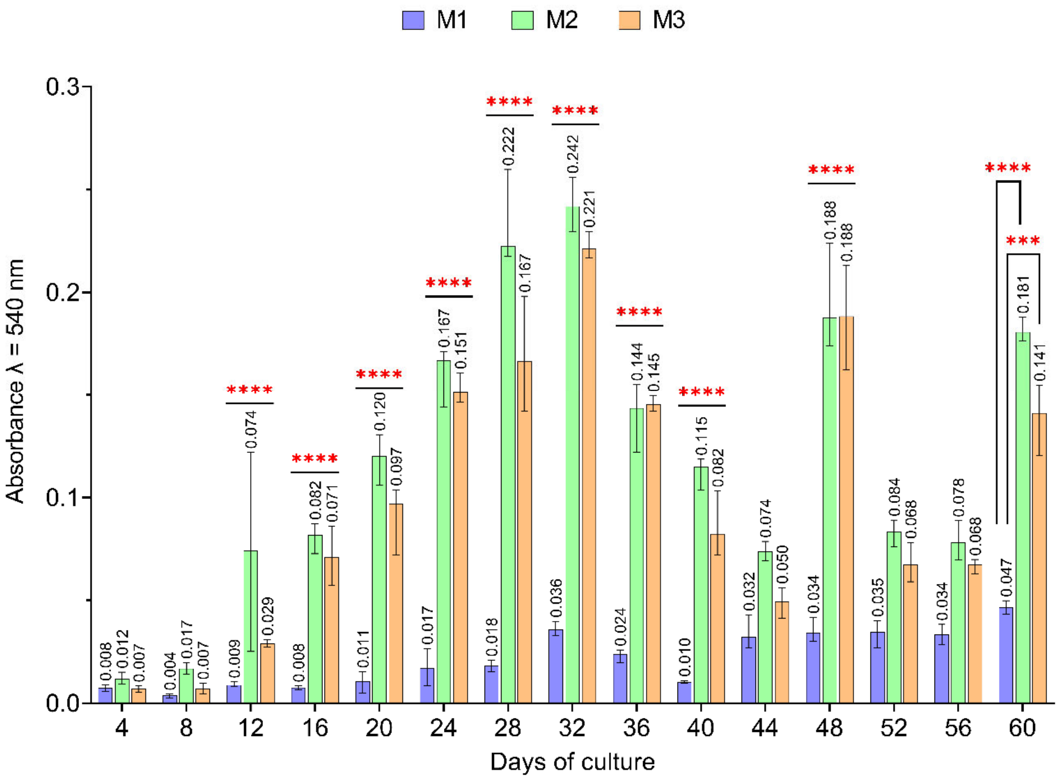

Figure S8. Fibroblasts ATCC CCL-1 colonisation of uncoated surgical meshes M1, M2 and M3. M1 (Adhesix™, BARD, New Providence, New Jersey, USA); M2 (Hermesh 4, Polhernia, Gdansk, Poland); M3 (Hermesh 8, Herniamesh® S.r.l. Chivasso, Italy); *** – high statistically significance ($p = 0.0006$); **** – very high statistically significance ($p < 0.0001$); whiskers show median with 95% of confidence interval. Full statistical details are shown in Table S8.

Table S8. Statistical comparisons of fibroblasts ATCC CCL-1 colonisation between uncoated surgical meshes M1, M2 and M3. M1 (Adhesix™, BARD, New Providence, New Jersey, USA); M2 (Hermesh 4, Polhernia, Gdansk, Poland); M3 (Hermesh 8, Herniamesh® S.r.l. Chivasso, Italy); Sign. Diff. – significant difference; Lv of diff. – level of difference; ns – no significant differences; *** - high statistically significance; **** - very high statistically significance.

Kruskal-Wallis test with post-hoc Dunne's modification; $\alpha = 0.05$						
**Day of culture**	**Sample**	**vs.**	**Sample**	**Sign. Diff.**	**Lv of diff.**	**Adjusted P**
**4**	M1	vs.	M2	No	ns	> 0.9999
	M1	vs.	M3	No	ns	> 0.9999
	M2	vs.	M3	No	ns	> 0.9999
**8**	M1	vs.	M2	No	ns	0.5708
	M1	vs.	M3	No	ns	> 0.9999
	M2	vs.	M3	No	ns	> 0.9999
**12**	M1	vs.	M2	Yes	****	< 0.0001

	M1	vs.	M3	No	ns	0.5112
	M2	vs.	M3	No	ns	0.4258
**16**	M1	vs.	M2	Yes	****	< 0.0001
	M1	vs.	M3	Yes	****	< 0.0001
	M2	vs.	M3	No	ns	> 0.9999
**20**	M1	vs.	M2	Yes	****	< 0.0001
	M1	vs.	M3	Yes	****	< 0.0001
	M2	vs.	M3	No	ns	> 0.9999
**24**	M1	vs.	M2	Yes	****	< 0.0001
	M1	vs.	M3	Yes	****	< 0.0001
	M2	vs.	M3	No	ns	> 0.9999
**28**	M1	vs.	M2	Yes	****	< 0.0001
	M1	vs.	M3	Yes	****	< 0.0001
	M2	vs.	M3	No	ns	> 0.9999
**32**	M1	vs.	M2	Yes	****	< 0.0001
	M1	vs.	M3	Yes	****	< 0.0001
	M2	vs.	M3	No	ns	> 0.9999
**36**	M1	vs.	M2	Yes	****	< 0.0001
	M1	vs.	M3	Yes	****	< 0.0001
	M2	vs.	M3	No	ns	> 0.9999
**40**	M1	vs.	M2	Yes	****	< 0.0001
	M1	vs.	M3	Yes	****	< 0.0001
	M2	vs.	M3	No	ns	> 0.9999
**44**	M1	vs.	M2	No	ns	0.2434
	M1	vs.	M3	No	ns	> 0.9999
	M2	vs.	M3	No	ns	> 0.9999
**48**	M1	vs.	M2	Yes	****	< 0.0001
	M1	vs.	M3	Yes	****	< 0.0001
	M2	vs.	M3	No	ns	> 0.9999
**52**	M1	vs.	M2	No	ns	0.0738
	M1	vs.	M3	No	ns	0.4994
	M2	vs.	M3	No	ns	> 0.9999
**56**	M1	vs.	M2	No	ns	0.2337
	M1	vs.	M3	No	ns	0.4748
	M2	vs.	M3	No	ns	> 0.9999
**60**	M1	vs.	M2	Yes	****	< 0.0001
	M1	vs.	M3	Yes	***	0.0006

	M2	vs.	M3	No	ns	> 0.9999

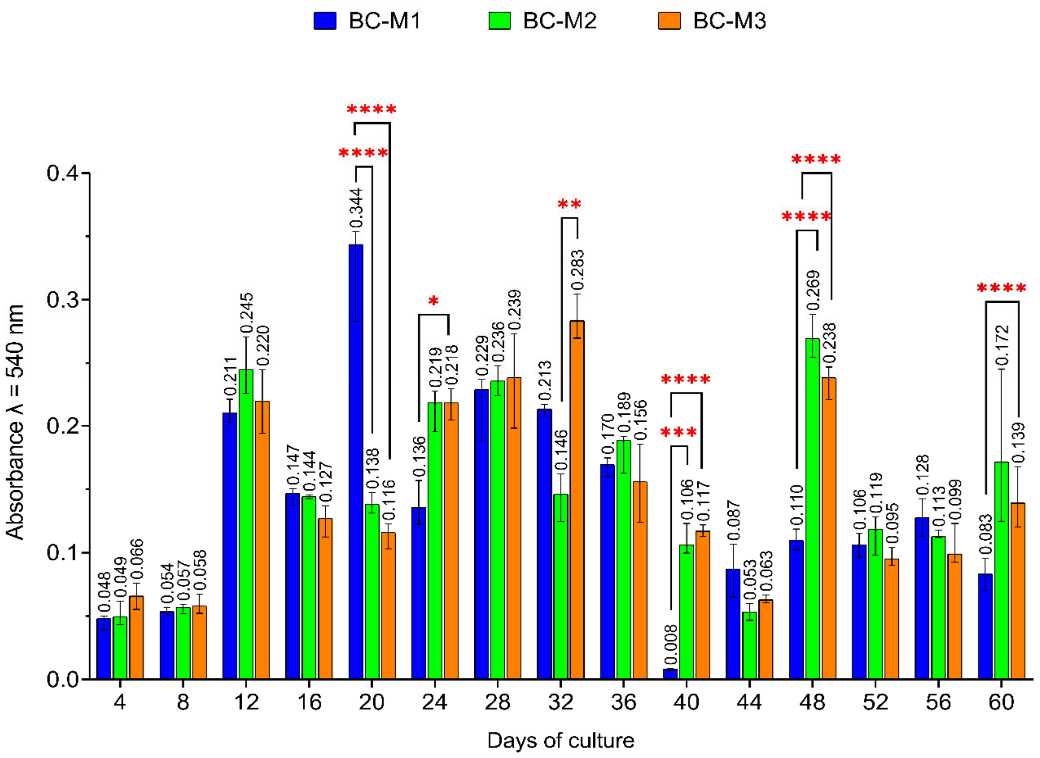

Figure S9. Fibroblasts ATCC CCL-1 colonisation of bacterial cellulose coated surgical meshes M1, M2 and M3. M1 (Adhesix™, BARD, New Providence, New Jersey, USA); M2 (Hermesh 4, Polhernia, Gdansk, Poland); M3 (Hermesh 8, Herniamesh® S.r.l. Chivasso, Italy); BC –bacterial cellulose; * – low statistically significance ($p = 0.0382$); ** – moderate statistically significance ($p = 0.0016$); *** – high statistically significance ($p = 0.0003$); **** – very high statistically significance ($p < 0.0001$); whiskers show median with 95% of confidence interval. Full statistical details are shown in Table S9.

Table S9. Statistical comparisons of fibroblasts ATCC CCL-1 colonisation between bacterial cellulose coated surgical meshes M1, M2 and M3. M1 (Adhesix™, BARD, New Providence, New Jersey, USA); M2 (Hermesh 4, Polhernia, Gdansk, Poland); M3 (Hermesh 8, Herniamesh® S.r.l. Chivasso, Italy); BC –bacterial cellulose; Sign. Diff. – significant difference; Lv of diff. – level of difference; ns – no significant differences; * - low statistically significance; ** - moderate statistically significance; *** - high statistically significance; **** - very high statistically significance.

Kruskal-Wallis test with post-hoc Dunne's modification; $\alpha = 0.05$						
**Day of culture**	**Sample (BC coated mesh)**	**vs.**	**Sample (BC coated mesh)**	**Sign. Diff.**	**Lv of diff.**	**Adjusted P**
**4**	M1	vs.	M2	No	ns	> 0.9999
	M1	vs.	M3	No	ns	> 0.9999
	M2	vs.	M3	No	ns	> 0.9999
**8**	M1	vs.	M2	No	ns	> 0.9999
	M1	vs.	M3	No	ns	> 0.9999
	M2	vs.	M3	No	ns	> 0.9999
**12**	M1	vs.	M2	No	ns	> 0.9999

	M1	vs.	M3	No	ns	> 0.9999
	M2	vs.	M3	No	ns	> 0.9999
**16**	M1	vs.	M2	No	ns	> 0.9999
	M1	vs.	M3	No	ns	> 0.9999
	M2	vs.	M3	No	ns	> 0.9999
**20**	M1	vs.	M2	Yes	****	< 0.0001
	M1	vs.	M3	Yes	****	< 0.0001
	M2	vs.	M3	No	ns	> 0.9999
**24**	M1	vs.	M2	No	ns	0.3815
	M1	vs.	M3	Yes	*	0.0382
	M2	vs.	M3	No	ns	> 0.9999
**28**	M1	vs.	M2	No	ns	> 0.9999
	M1	vs.	M3	No	ns	> 0.9999
	M2	vs.	M3	No	ns	> 0.9999
**32**	M1	vs.	M2	No	ns	> 0.9999
	M1	vs.	M3	No	ns	> 0.9999
	M2	vs.	M3	Yes	**	0.0016
**36**	M1	vs.	M2	No	ns	> 0.9999
	M1	vs.	M3	No	ns	> 0.9999
	M2	vs.	M3	No	ns	> 0.9999
**40**	M1	vs.	M2	Yes	***	0.0003
	M1	vs.	M3	Yes	****	< 0.0001
	M2	vs.	M3	No	ns	> 0.9999
**44**	M1	vs.	M2	No	ns	> 0.9999
	M1	vs.	M3	No	ns	> 0.9999
	M2	vs.	M3	No	ns	> 0.9999
**48**	M1	vs.	M2	Yes	****	< 0.0001
	M1	vs.	M3	Yes	****	< 0.0001
	M2	vs.	M3	No	ns	> 0.9999
**52**	M1	vs.	M2	No	ns	> 0.9999
	M1	vs.	M3	No	ns	>0.9999
	M2	vs.	M3	No	ns	> 0.9999
**56**	M1	vs.	M2	No	ns	> 0.9999
	M1	vs.	M3	No	ns	> 0.9999
	M2	vs.	M3	No	ns	> 0.9999
**60**	M1	vs.	M2	Yes	****	< 0.0001
	M1	vs.	M3	No	ns	0.0550

	M2	vs.	M3	No	ns	> 0.9999

Table S10. Statistical data to „Bacterial cellulose water content determination" section. Sign. Diff. – significant difference; Lv of diff. – level of difference; **** - very high statistically significance.

Kruskal-Wallis test with post-hoc Dunne's modification; $\alpha = 0.05$					
**Sample**	**vs.**	**Sample**	**Sign. Diff.**	**Lv of diff.**	**Adjusted P**
wet BC	vs.	dry BC	Yes	****	< 0.0001
mesh	vs.	BC on mesh	Yes	****	< 0.0001

Table S11. Statistical data to „Modified disc diffusion method" section. MIC – minimal inhibitory concentration for gentamicin against *Staphylococcus aureus* ATCC 33591 determined in presented research (0,47 μg/ml); GS – concentration of gentamycin in gentamycin sponge (4,0 mg/ml); BC –bacterial cellulose; Sign. Diff. – significant difference; Lv of diff. – level of difference, ns – no significant differences; ** - moderate statistically significance; *** - high statistically significance.

Kruskal-Wallis test with post-hoc Dunne's modification; $\alpha = 0.05$					
**Sample**	**vs.**	**Sample**	**Sign. Diff.**	**Lv of diff.**	**Adjusted P**
MIC-mesh	vs.	MIC-BC-mesh	No	ns	> 0.9999
MIC-mesh	vs.	GS-mesh	Yes	**	0.0084
MIC-BC-mesh	vs.	GS-BC-mesh	Yes	***	0.0003
GS-mesh	vs.	GS- BC-mesh	No	ns	0.3319

Table S12. Average fibroblasts quantity (measures as an absorbance with neutral red, λ = 490 nm) and a fold of difference in average fibroblasts quantity. M1 (Adhesix™, BARD, New Providence, New Jersey, USA); M2 (Hermesh 4, Polhernia, Gdansk, Poland); M3 (Hermesh 8, Herniamesh® S.r.l. Chivasso, Italy); BC-M1/M2/M3 – described meshes coated with bacterial cellulose.

days of culture	average fibroblasts quantity (measures as an absorbance with neutral red, λ = 490 nm)						a fold of difference in average fibroblasts quantity		
	M1	BC-M1	M2	BC-M2	M3	BC-M3	BC-M1 vs M1	BC-M2 vs. M2	BC-M3 vs. M3
**4th**	0.0073	0.0520	0.0123	0.0524	0.0067	0.0649	7.1	4.3	9.6
**8th**	0.0035	0.0549	0.0174	0.0589	0.0099	0.0594	15.7	3.4	6.0
**12th**	0.0104	0.2178	0.0912	0.2464	0.0316	0.2268	20.9	2.7	7.2
**16th**	0.0078	0.1439	0.0828	0.1539	0.0706	0.1284	18.4	1.9	1.8
**20th**	0.0199	0.3327	0.1069	0.1322	0.0876	0.1182	16.7	1.2	1.3
**24th**	0.0192	0.1440	0.1822	0.2090	0.1622	0.2363	7.5	1.1	1.5
**28th**	0.0254	0.2165	0.2339	0.2126	0.1732	0.2327	8.5	0.9	1.3
**32nd**	0.0351	0.2148	0.2302	0.1577	0.2138	0.2878	6.1	0.7	1.3
**36th**	0.0230	0.1549	0.1416	0.1760	0.1461	0.1610	6.7	1.2	1.1
**40th**	0.0105	0.0077	0.1100	0.1144	0.0852	0.1164	0.7	1.0	1.4
**44th**	0.0320	0.0891	0.0730	0.0532	0.0493	0.0623	2.8	0.7	1.3
**48th**	0.0388	0.1129	0.1867	0.2593	0.1915	0.2500	2.9	1.4	1.3
**52nd**	0.0347	0.0945	0.0845	0.1171	0.0728	0.0929	2.7	1.4	1.3
**56th**	0.0372	0.1119	0.0819	0.1174	0.0761	0.1089	3.0	1.4	1.4
**60th**	0.0424	0.0877	0.1669	0.1977	0.1303	0.1471	2.1	1.2	1.1
**4th vs. 60th**	5.8	1.7	13.6	3.8	19.3	2.3			
**4th**							7.1	4.3	9.6
**60th**							2.1	1.2	1.1

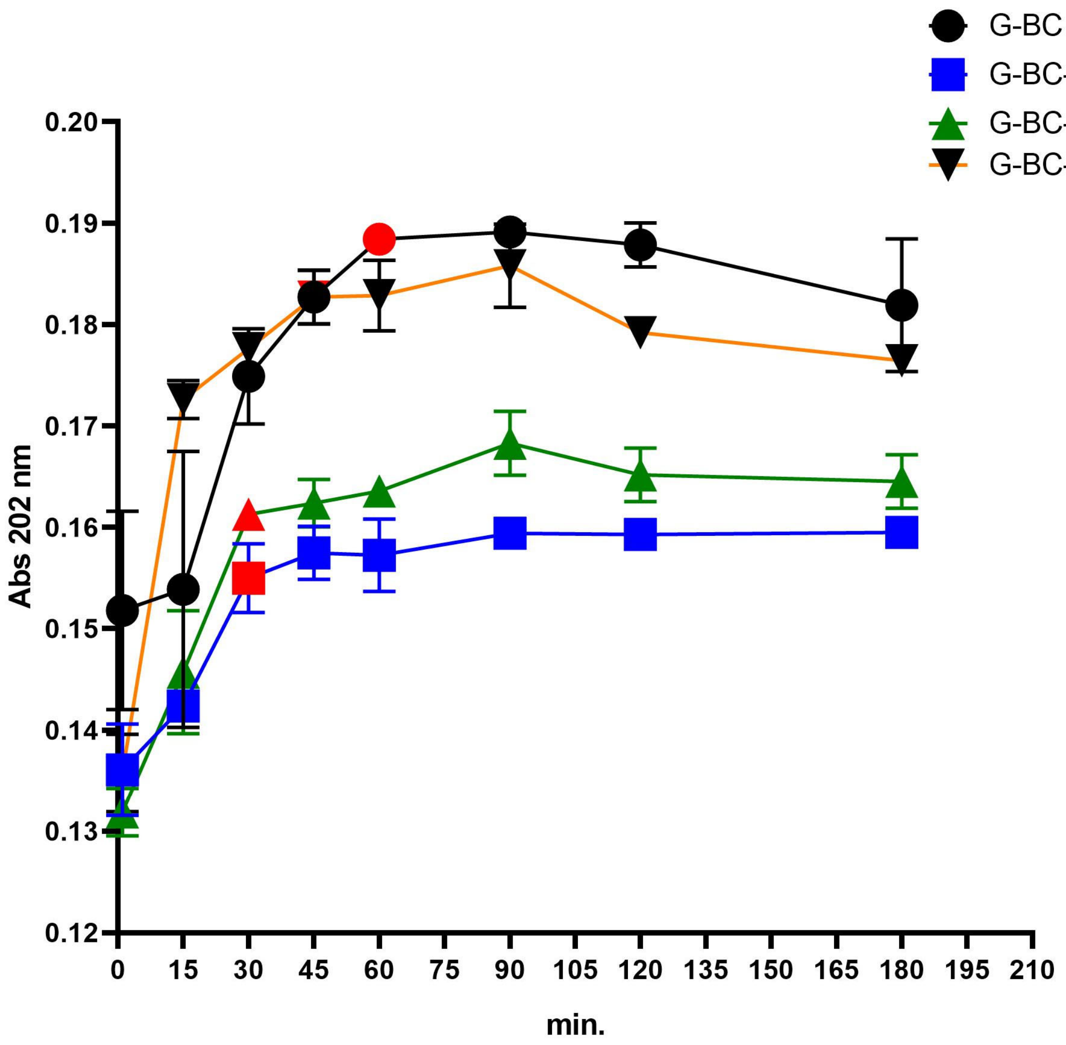

Figure S10. The release profile of gentamycin from the BC and BC-coated M1, M2, M3 meshes. Red colouring indicates the specific time points, in which the concentration of released gentamicin stopped to increase. G-BC-M1/M2/M3 – gentamicin saturated bacterial cellulose coated meshes M1, M2, M3, respectively.

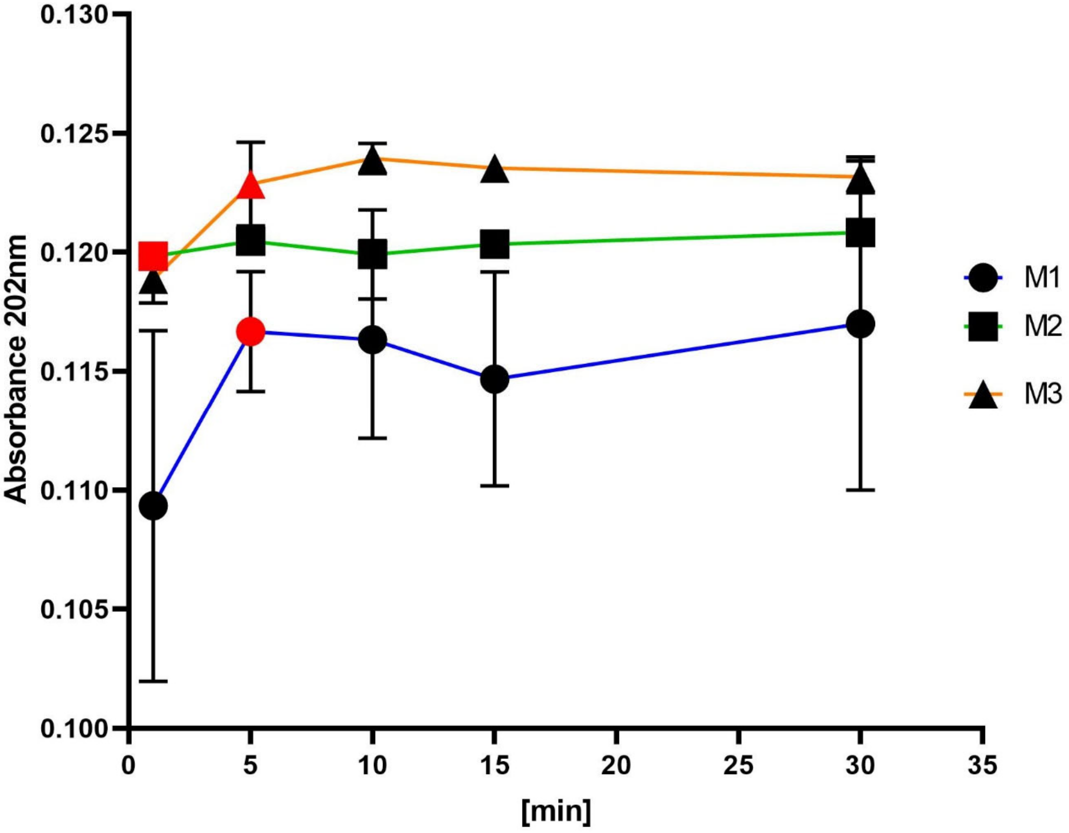

Figure S11. The release profile of gentamycin from the uncoated M1, M2, M3 meshes. Red colouring indicates the specific time points, in which the concentration of released gentamicin stopped to increase. M1, M2, M3 – uncoated meshes.

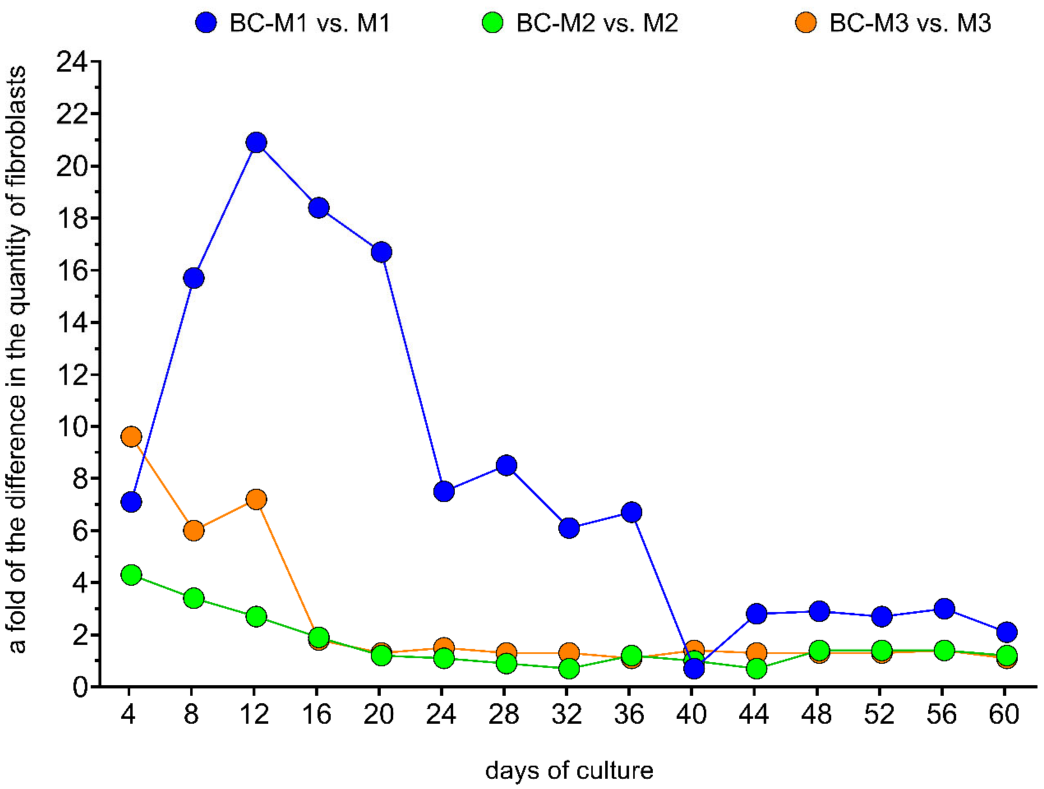

Figure S12. Comparison of average fibroblasts quantity between bacterial cellulose coated and uncoated surgical meshes shows as a fold of difference in average fibroblasts quantity. M1 (Adhesix™, BARD, New Providence, New Jersey, USA); M2 (Hermesh 4, Polhernia, Gdansk, Poland); M3 (Hermesh 8, Herniamesh® S.r.l. Chivasso, Italy); BC-M1/M2/M3 – described meshes coated with bacterial cellulose.
